# Multiscale and re-entrant surface analysis of multi jet fusion surfaces and the effect of postprocessing via sandblasting

**DOI:** 10.1038/s41598-025-27015-8

**Published:** 2025-12-03

**Authors:** Patryk Mietliński, François Berkmans, Katarzyna Peta, Tomasz Bartkowiak, Maxence Bigerelle, Bartosz Gapiński, Michał Wieczorowski

**Affiliations:** 1 Poznań University of Technology, Poznań, Poland; 2 University Polytechnic Hauts-De-France, Valenciennes, France

**Keywords:** Surface analysis, Computed tomography, MJF, 3D printing, Additive technology, Post-processing, Re-entrants surface, Engineering, Materials science

## Abstract

The growing use of Multi Jet Fusion (MJF) 3D printing in industry brings challenges related to surface quality and post-processing effects. This study presents a comprehensive analysis of surface geometry changes in MJF-printed parts before and after sandblasting. A suitable abrasive material was selected and tested, followed by chemical composition analysis to confirm the absence of abrasive residues on the surfaces. Glass beads with a radius of 37.5 to 70 μm were used for sandblasting. Micro-computed tomography (µCT) was used as the only technique capable of capturing complex surface topography, including re-entrant features, both pre- and post-processing. Surface characterization was performed following ISO 25,178 roughness parameters and area-scale analysis for a total of 30 surfaces. A proprietary algorithm was also implemented to quantify re-entrant features. Results showed significant changes in surface texture due to sandblasting, including reduced surface roughness and enhanced uniformity. For instance, *Sa* decreased from 22.3 μm to 14.6 μm, and *Sp* dropped by over 30%. Multiscale analysis revealed a 50% reduction in surface fractal complexity (*Asfc*) and a significant decrease in fine-scale geometric variability. An algorithm for detecting reentrant features was developed, in which original indicators for their quantitative occurrence were introduced: r₁ and r₂. On average r₂ values dropped from 60% to 13%, and r₁ from 260% to 135%. ANOVA confirmed statistically significant differences (*p* < 0.001) between pre- and post-processing states (MSR coefficients for *Srel* and *Asfc* parameters were used). Additionally, a statistical analysis was performed, which showed significant and large impact of post-processing on the geometric character of the surface. The study demonstrates that µCT is uniquely capable of isolating unsintered powder and characterizing complex surface features. Sandblasting significantly alters surface geometry and must be accounted for during design, especially in applications requiring precise topographical control.

## Introduction

Additive Manufacturing (AM) technology, more commonly known as 3D printing, involves the incremental production of components layer by layer. This method contrasts with conventional technological methods, where the formation of a given component occurs through subtractive processing. Additive technologies encompass various ways of shaping geometry. In this article, the authors will address issues related to Multi Jet Fusion. To understand the significance of individual aspects of MJF, the article will expand upon and discuss the technique itself, its underlying principles, and conditions in further detail.

HP MJF 3D printing involves the layered fusion of plastic powders. Properly prepared and preheated powder is applied within the working area using a roller. Subsequently, a precise printing head applies two types of agents to assist the spatial printing process. The first of these agents – the fusing agent – is dosed within the model area (where the part geometry is replicated), and its properties lead to increased radiation absorption. The second agent – detailing agent – is applied at the external contours of the elements to facilitate easier separation of unmelted powder and improve the accuracy of replication. The detailing agent helps ensure clear part edges for achieving significantly finer details. Then, energy is uniformly applied to the applied powder layer all at once. This makes MJF a much faster method than laser-based processes, which fuse point by point as the laser beam moves. Areas covered with fusing agent, along with the volume of powder it was applied to, are melted. Meanwhile, the powder coated with the detailing agent remains loose, defining the precise boundaries of the part. This process repeats layer by layer until the final part is formed. Agents are applied with a resolution of 1200 dpi in the X/Y axes. In MJF, new powder layers are spread while the previous ones still retain residual heat, which helps the fusing agents and infrared energy to fully melt the particles and bond layers together.

**Fig. 1:**
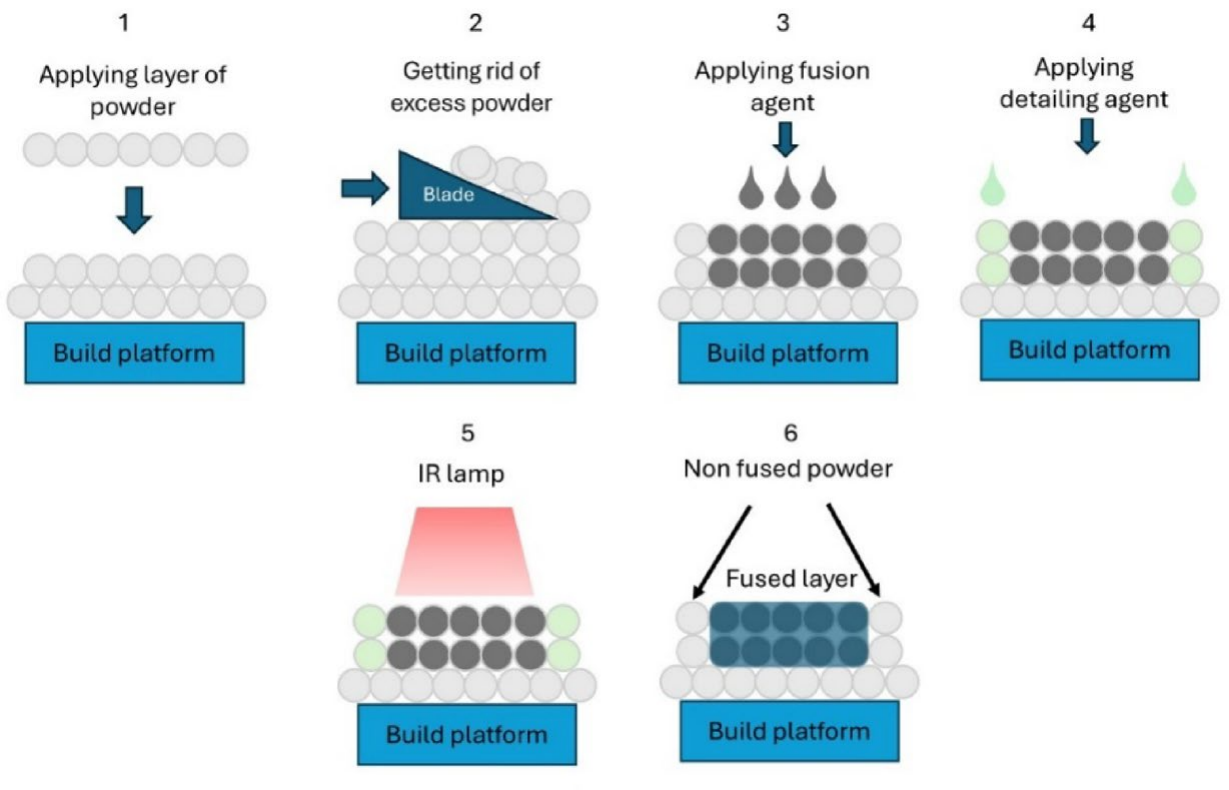
Diagram of individual stages of MJF printing.

This improved interlayer fusion increases part strength and enables finer detail reproduction^1^. The principles of MJF operation and its individual stages are depicted in Fig. 1.

The MJF technology, as a relatively new 3D printing technique, has only recently entered the market. MJF was developed internally at HP in 2013–2014, publicly unveiled in the fall of 2014, and commercially launched in 2016. However, it is steadily gaining traction among scientists and is consequently finding applications in numerous manufacturing sectors. The primary areas of application for MJF-printed components include medicine^2–4^, electronics^5,6^ and machine parts^7–11^. Elements produced using MJF technology always require post-processing. It becomes imperative to eliminate remnants of unmelted powder lingering after the manufacturing process. This is attributed to the application of detailing agent in its area, which, when combined with the powder, aims to delineate the boundaries of the emerging geometry. It adheres to the walls and openings of the component in accordance with the principles of MJF printing. Although there are various post-processing options for MJF^12–14^, the most common is sandblasting^15^.

Each of the aforementioned processes influences the geometric characteristics of the surface to varying degrees. However, given the potential and current applications, as well as the drawbacks, advantages, essence, and evolution of MJF technology, surface quality emerges as a pivotal factor for the future development of potential areas implementing MJF printing. Furthermore, it is noteworthy that issues concerning surface form impact mechanical properties^16^, the part’s propensity for crack propagation^17^, contact mechanics^18^ and fluid flow resistance^19^. Considering surface alterations after post-processing, it is essential to evaluate how the surface changes during cleaning of the samples.

Assessing the surface both before and after the removal of unmelted powder enables a clear measurement of the magnitude and nature of these changes. In this study, we opted to examine surfaces post-sandblasting processing. The rationale for selecting this method is its status as one of the most commonly employed finishing operation for MJF elements and its minimal impact on surface geometric characteristics^20,21^. Hence, it is logical and consistent to investigate this method to bolster and align current research with the broader community of scientists and engineers for the further advancement of MJF technology. The authors are unaware of any other scientific research that comprehensively addresses issues related to surface alteration due to sandblasting post-MJF production. Additionally, surface studies will be complemented by chemical and analyses of the produced samples.

The article studies the impact of sandblasting on the comprehensive geometric properties of the surface elements produced by MJF. This, in turn, allows the design of technologies and production processes tailored to specific applications and the desired properties of the elements necessary at the final stage of production. In this way, the following dependencies are directly determined: surface - postprocessing - surface. Computed tomography is not commonly employed for surface measurements. Bartkowiak et al.^22^, Thompson et al.^23^ and Key et al.^24^ used it in their studies. This enabled them to visualize surfaces that would have otherwise been impossible to observe using alternative measurement methods. In addition, multiscale analysis was used. Multiscale parameters reliably complement conventional parameters in the characterization of additively manufactured surfaces^25^.

Additionally, the article includes the analysis that may potentially influence the obtained results, such as the size of the abrasive and the chemical characteristics of the samples.

## Review of MJFed surface research

Surface metrology aimed to study both the relationship between a surface condition and a manufacturing process, as well as surface functionality^26,27^. The case of studying Multi Jet Fusion, although a relatively new process, has naturally attracted researchers who studied the surface topographies of industrial processes. Indeed, evaluating the surface of components is a crucial factor in assessing the quality of printing, as wear, fatigue, and corrosion always begin from the surface of parts^28^.

To account for the studies that have already been conducted on surface topography, we can separate them into two categories: firstly, the measurement devices used; secondly, the roughness parameters selected.

For MJF, the study by Tey et al.^29^ presented the use of a laser confocal microscope. The use of White light interferometry was presented in the study of Chand et al.^30^. Tactile profilometer solution was also used for 2D and 3D measurements in Binnur et al.^31^ and in Tasch et al.^32^. Atomic force microscope was also used but in the special case of a composite material using aramid fibers and PA12. The researchers focused the roughness analysis on the aramid fibers and not PA12^33^. he use of conventional optical microscopes for quantitative surface topography analysis is uncommon, as they are typically limited to qualitative surface inspection. However, Alomarah et al.^34^ demonstrated that optical microscopy can still provide useful insights for general surface condition assessment, even though it cannot produce quantitative 3D roughness data comparable to profilometry or interferometry. The issue is that a profilometer can generate a 3D map as a point cloud to quantify roughness parameters, which is not possible with photographs from optical microscopes and SEM^33^.

The use of a particular measurement device is directly linked to the scale and nature of the observed phenomenon. For comparison, the most used devices in metal additive manufacturing are focus variation microscopes (i.e., like our study) and confocal microscopes^35^. Coherence scanning interferometry is less commonly used for measuring additive manufacturing (AM) surfaces due to challenges posed by their highly irregular nature, such as local slope and vertical roughness scale. Similarly, atomic force microscopy (AFM) is rarely used for AM metallic surfaces because of limitations in vertical measurement range and the risk of damaging the stylus. Although many studies still employ conventional 2D imaging techniques such as SEM or optical microscopy for qualitative assessment, an increasing number of works use advanced 3D profilometry methods and multi-scale roughness parameters to quantify AM surfaces. Most researchers characterizing AM surfaces rely on conventional 2D imaging techniques, primarily scanning electron microscopy and optical microscopy, for qualitative visualization, whereas quantitative roughness evaluation typically employs confocal or focus variation microscopes^36,37^.

The relevant parameters highlighted in a study of the surface topography of objects manufactured using MJF depend on the materials used and the way the surface was modified during post-processing. We note that multi-scale analyses of the surfaces of Multi Jet Fusion elements are rarely studied, which is a strong point of our study. In the study of Tey et al.^29^, the topographical analysis is very basic and is limited to the use of the 2D parameter *Ra*, which determines the average of absolute heights relative to the mean plane over a 2D profile. A comparison of *Ra* values can indeed provide indications of increased roughness between surface A and surface B. However, two similar *Ra* values can represent very different topographies. In a study analysing mechanical performance other parameters should be studied. A combination of 2D and 3D parameters are used for each surface of parts printed using MJF in the study Binnur et al.^31^ The parameters *Ra* and *Rq*, as well as *Sa* and *Sq* (root mean square of ordinate values), are compared. However, the limitations of the previous study remain the same, as *Ra* and *Rq* parameters do not fully capture the three-dimensional complexity of MJF surfaces. Nevertheless, considering that the article primarily focused on dimensional accuracy rather than functional surface behavior, these two parameters provided sufficiently consistent results to support the authors’ conclusions. In fractography study on MJF such as Tasch et al.^32^ the fractal dimension aka *Sfd* parameter in ISO 25,178^38^ is supposed to be considered, unfortunately this study lacks of depths considering analysis of surface topography. Because the study only provides an average 2D profile roughness of the surface (*Ra*), and not an in-depth analysis of the fractal dimension of the fracture zone from the tests. The last study present interesting parameter than can describe better the micro geometry of the surface^39^. Parameters such as *Sq Ssk* and *Sku* are sensitive to the distribution and asymmetry of surface features. In the cited studies, these parameters allowed researchers to detect subtle differences in layer consolidation and powder spreading, depending on the part’s position on the build platform. As we can see in this section, more in-depth analyses in the study of surface topography in MJF can enrich the literature, particularly with regard to multi-scale analysis.

## Methods

### Manufacturing of the samples

**Fig. 2:**
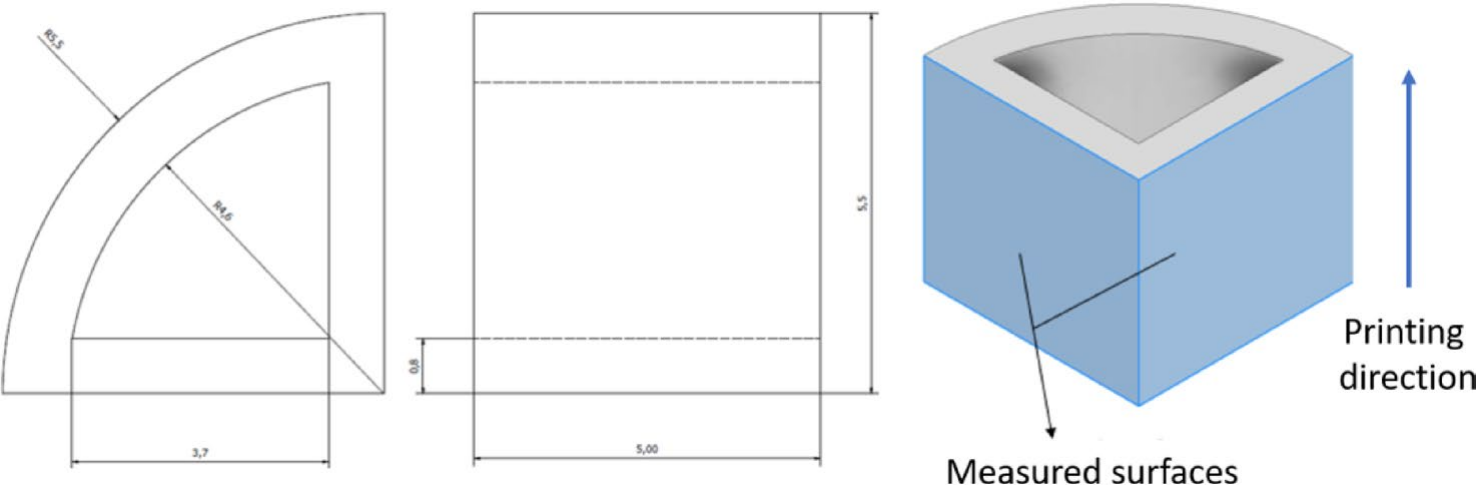
Dimensions and model of the manufactured MJF samples (values ​​are given in mm).

To conduct surface analysis, 30 identical samples were prepared. The samples had the shape of a quarter cylinder with a radius of 5.5 mm and a through hole inside. The thickness of the wall was 0.8 mm. The height of the element was set at 5 mm. For surface analysis, two external surfaces at a 90° angle were selected. The dimensions and model indicating the measured surfaces are shown in Fig. 2.

The samples were printed using an HP MJF 5200 printer. The working volume was.

380 × 284 × 380 mm with a maximum print speed of 5058 cm/h. The layer thickness was 80 μm with a print resolution of 1200 dpi. All samples were printed with the same print parameters vertically relative to the printing platform – the base surface of the quarter cylinder was parallel to the table (Table 1).

**Table 1:** Parameters of the printer on which the samples were made.

MJF printer characteristics	
Printer	HP MJF 5200
Building volume	380 × 284 × 380 mm^3^
Building speed	up to 5058 cm/h
Layer thickness	80 μm
Print resolution (x, y)	1200 dpi

Polyamide 12 (PA12) material powder was used to make the samples. The powder diameter was 60 μm. The power melting temperature was 187 °C, while the bulk density was 1.01 g/cm^3^. Powder parameters are presented in Table 2^40^.

**Table 2:** Powder parameters.

MJF powder properties			
	Powder size [µm]	Powder melting point [°C]	Bulk density g/cm^3^
PA12 powder	60	187	1.01

### Surface topography

For measurement and analysis, two perpendicular surfaces of each sample were selected as shown in Fig. 2. This yielded a total of 60 test surfaces. The measurement field was 2.5 mm x 2.5 mm. This approach enabled the elimination of human errors associated with the post-processing operation, thus ensuring the most accurate and reliable representation of the surface analysis results. The Table 3 shows a summary of the number of samples and a description of the tested surfaces.

**Table 3:** Characteristics of the list of sample designations and quantities, and surface areas.

List of samples and surfaces				
	Sample name	Surface name	Surface name	Amount surfaces
	1	Sample 1 surf_1	Sample 1 surf_2	2
	2	Sample 2 surf_1	Sample 2 surf_2	2
	…	…	…	…
	30	Sample 30 surf_1	Sample 30 surf_2	2
Total amount	30	30	30	60

Surface measurements were conducted in two stages. The initial stage entailed measuring the surface prior to the post-processing step of sandblasting. Subsequently, in the second stage, measurements focused on assessing the surface post-processing. Analyzes of measurement data included comparison of conventional^38^ and multi-scale surface characterization parameters^41^.

**Fig. 3:**
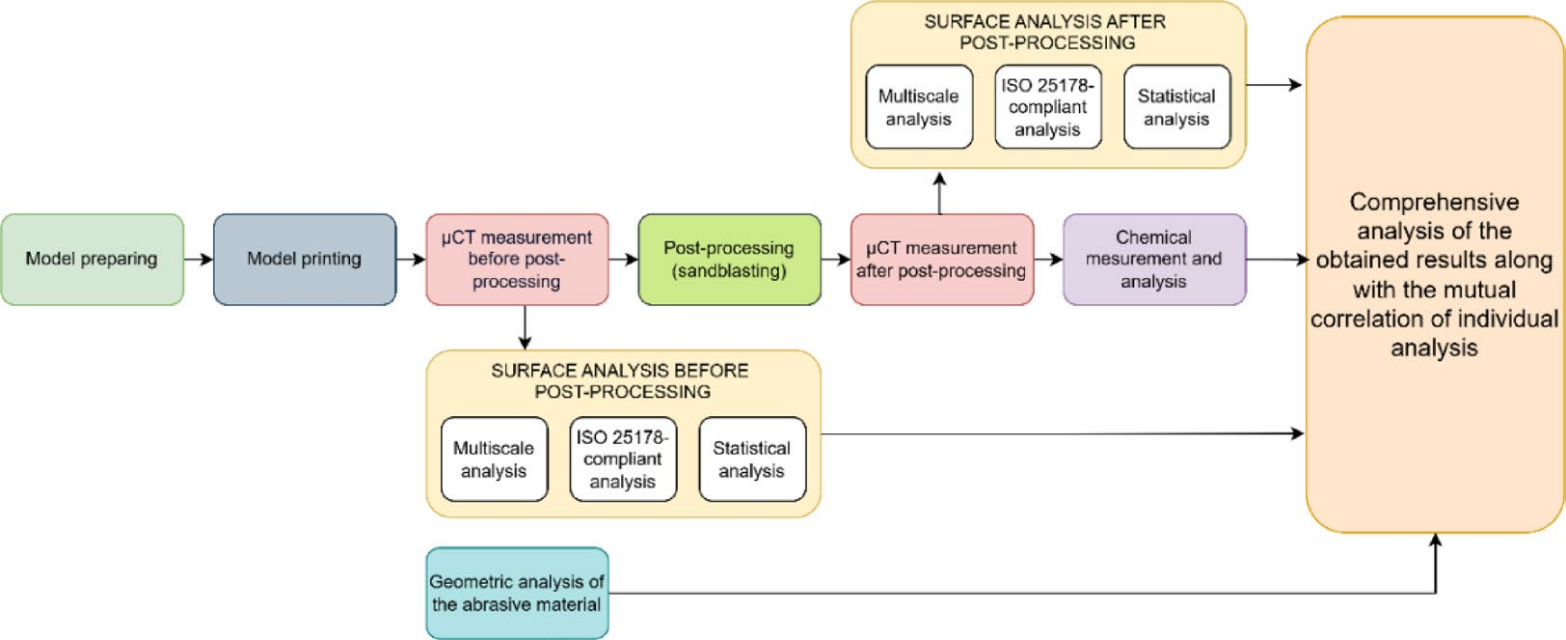
Scheme of the study conducted.

As outlined in Sect. 1, components manufactured using MJF technology are enveloped in unmelted powder following the printing process. Consequently, traditional surface measurement techniques would be ineffective prior to powder removal, as it conceals the surfaces of the printed elements throughout the sample area. Micro computed tomography emerges as the sole method capable of capturing the hidden geometry. Thus, in Stage 1, it was chosen as the exclusive method to visualize the concealed surface. The research methodology is depicted in Fig. 3.

#### X-ray micro computed tomography

Computed tomography is considered a non-destructive diagnostic method that uses X-ray radiation. It allows examinining the parts with intricate geometric shapes and analyzing of their internal structures that may not be visible using other measurement methods^42^.

The v|tome|x s240 computed tomography scanner (manufactured by Waygate Technologies / GE Sensing & Inspection Technologies GmbH) was used for the measurements. The parameters were specified as follows: voltage set at 130 kV; current at 120 µA; exposure time for each image at 350 ms; voxel size at 3.5 μm; and a total of 2000 images were captured. Tomographic analyses were conducted using Volume Graphics MAX 2.2 software (Heidelberg GmbH, Germany).

Through the application of this method and the selection of the aforementioned parameters, it became possible to measure the samples and subsequently differentiate between the melted and loose powder. Consequently, a model was generated, and specific surfaces were isolated for further analysis.

**Fig. 4:**
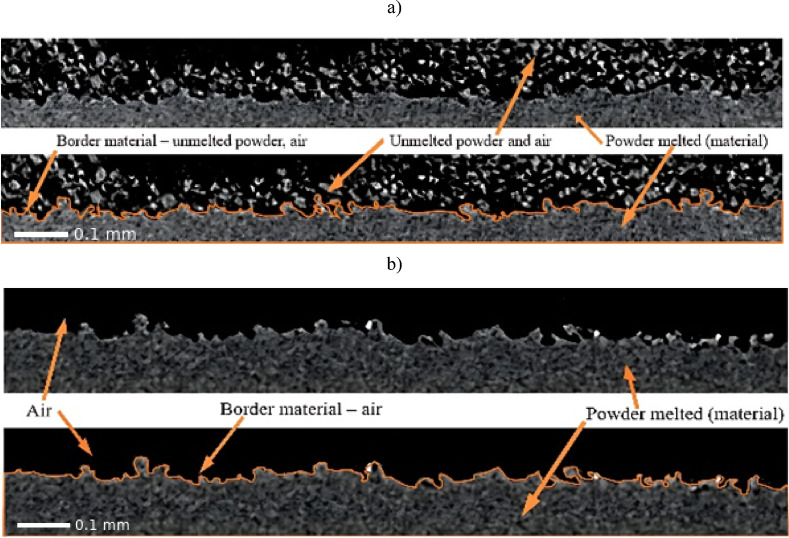
View of tomographic images of samples made with MJF technology: (**a**) before post-processing; (**b**) after post-processing *b)*

Tomographic measurements detect density variations between the material and surrounding air. Denser regions absorb more radiation, allowing X-ray images to reveal structural differences and enable 3D model reconstruction. In this study, distinguishing melted from unmelted powder was crucial. This separation was facilitated by the fusion agent applied during printing, which introduces substances such as soot and ethylene glycol into the melted regions^35^. Conversely, loose powder - the unmelted fraction that forms a protective layer on the surface - lacks these chemical compounds. Notably, the density of PA12 - the printing powder material - is 1.01 g/cm^3^, while the density of soot ranges from 1.7 to 1.9 g/cm^3^, and the density of triethylene glycol is 1.1 g/cm^3^. Consequently, melted powder fractions exhibit higher density than loose powder fractions, devoid of soot and triethylene glycol. This facilitated the identification of density discrepancies in the material on tomographic images and the digital separation of melted powder from unmelted powder. Figure 4a depicts a tomographic image before post-processing, showcasing both unmelted and melted powder along with the demarcation of the boundary between these regions. Conversely, Fig. 4b displays a tomographic image after post-processing, where loose powder is absent due to its removal.

a)

#### Multiscale analysis – area-scale method

The characterization and visualization of surface topography depends on the scale of observation^43^. Therefore, conventional topographic parameters (ISO 25178-2:2012)^38^ characterize surfaces only in a narrow range of scales. Height parameters are dedicated to larger scales, while hybrid parameters are more reliable at finer scales. The advantage of multiscale geometric parameters^41^ is their ability to characterize surfaces in a wide range of scales, from sampling distance to field of view. Multiscale geometric analyzes (aka scale-sensitive fractal analyses) developed by Brown et al.^44^ draw on Mandelbrot’s foundations of fractal shapes^45^. These analyzes characterize topographies using relative area (*Srel*), relative length (*RL*), area-scale complexity (*Asfc*), length-scale complexity (*Lsfc*). *Srel* and *Asfc* are components of area-scale analysis and describe the 3D surface area, while RL and Lsfc are related to length-scale analysis and characterize the 2D profile of the surface. *Srel* / *RL* represent the ratio of the calculated area/length to the nominal area/length. *Srel* and RL are related to the exercises of tiling the surface with tiles/length segments of different sizes, related to scales. *Asfc* and *Lsfc* are calculated from logarithmic plot of *Srel* or RL versus scale, equal to the product of -1000 and the slope of this plot^46^.

The relative area (*Srel*) and relative length (*RL*) are defined as:

$${S_{rel}}\left( \lambda \right)=\frac{{A\left( \lambda \right)}}{{{A_0}}}$$

$$RL\left( \lambda \right)=\frac{{L\left( \lambda \right)}}{{{L_0}}}$$

where *A*(*λ*) and L(λ) denote the measured surface area and profile length at a given scale λ, while.

*A*_*0*_ and *L*_0_ represent the nominal (projected) area and length, respectively.

The area-scale complexity *(Asfc*) and length-scale complexity (*Lsfc*) are derived as the negative slopes of the logarithmic plots of *Srel* or *RL* versus scale, according to:

$$Asfc= - 1000 \times \frac{{d\log {S_{rel}}}}{{d\log \lambda }}$$

$$Lsfc= - 1000 \times \frac{{d\log RL}}{{d\log \lambda }}$$

Both area-scale and length-scale analyses are reliable for characterizing isotropic and irregular surfaces^47^. In this study, area-scale analyses were chosen, which are adequate for describing irregular surfaces with randomly distributed reentrants.

#### Conventional analysis based on ISO parameters

In this study, the authors used a set of areal surface texture parameters to describe a series of measured samples (Table 4). Nine parameters (*Sq*,* Sa*,* Sp*,* Sv*,* Ssk*,* Sku*,* Sz*,* Sdq*,* Sdr*) defined in the ISO 25,178 standard were taken into account^30^. The parameters were selected based on a preliminary analysis of surface topography results in such a way that, according to their definitions and assumptions, they would best reflect the nature of surface changes resulting from post-processing and comprehensively describe the surface topography both before and after sandblasting. Areal surface parameters do not distinguish between waviness and roughness. Therefore, it is always necessary to specify the filtering method used and which surface the parameter refers to. In this study, roughness parameters were calculated for the S-L surface, using Gaussian filtering with a nesting index value of 250 μm.

**Table 4:** ISO 25,178 surface parameter.

Standard	Parameter group	Parameter symbol
ISO 25,178	Height	*Sq*,* Ssk*,* Sku*,* Sp*,* Sv*,* Sz*,* Sa*
	Hybrid	*Sdq*,* Sdr*
	Functional	*Smr*,* Smc*,* Sxp*
	Spatial	*Sal*,* Str*,* Std*
	Functional (volume)	*Vm*,* Vv*,* Vmp*,* Vmc*,* Vvc*,* Vvv*
	Feature	*Spd*,* Spc*,* S10z*,* S5p*,* S5v*,* Sda*,* Sha*,* Sdv*,* Shv*

The surface roughness parameters listed in Table 4 were subjected to mutual correlation analysis, as well as an assessment of their changes resulting from sandblasting. This allowed for a comprehensive investigation of the impact of post-processing on the geometric characteristics and topography of the surface. Additionally, the results were compared with the findings from the multiscale surface analysis and the implications of the presence of re-entrant features. Altogether, this enabled the examination of key surface changes occurring after the critical removal of unsintered powder following the manufacturing process.

#### Re-entrant features

**Fig. 5:**
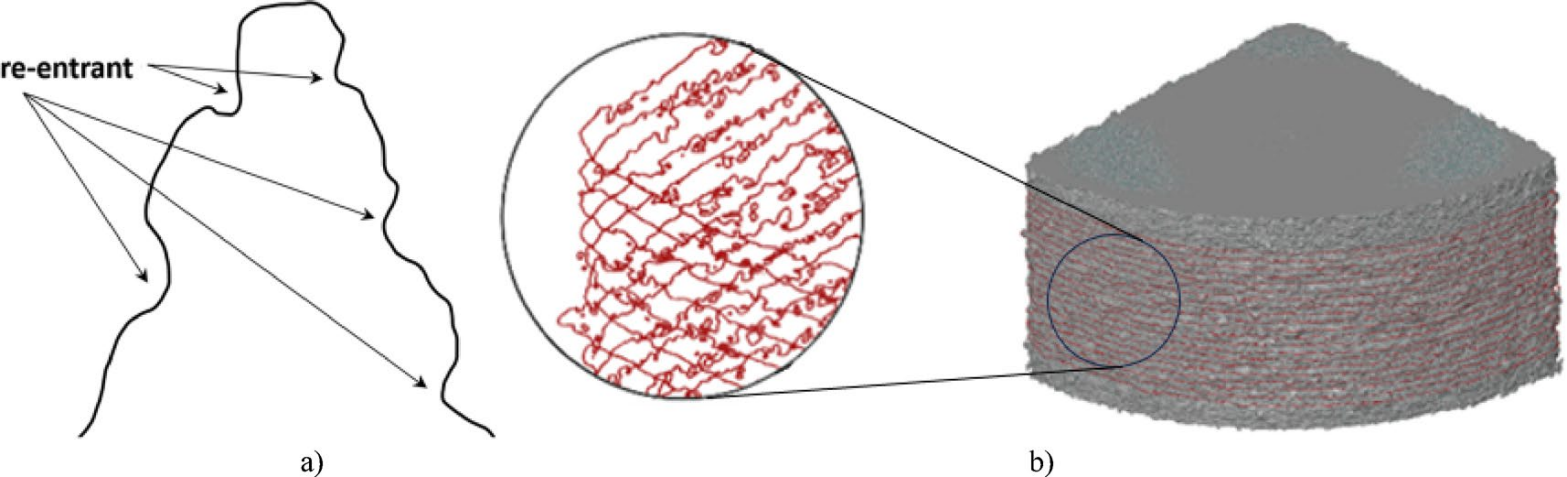
(**a**) evident re-entrant features; (**b**) view of re-entrants - model obtained from the µCT measurement, additionally profile view, samples made with the PBF technique.

The surface formed using PBF printing techniques is characterized by the creation of re-entrant features. Re-entrant features are geometric surface structures^48^. Their formation and shape are related to the form of the powder itself, which has a spherical shape^49^. So far, testing has been conducted using contact and optical methods. However, with the advancement of manufacturing techniques, the structure of shaped surfaces has begun to exhibit unfamiliar shapes and characteristics. This issue has become apparent particularly in PBF technologies. Traditional surface profiles no longer suffice, as surfaces now exhibit multiple elevations for a single abscissa. This phenomenon is referred to as a re-entrant surface (Fig. 5a, b).

**Fig. 6:**
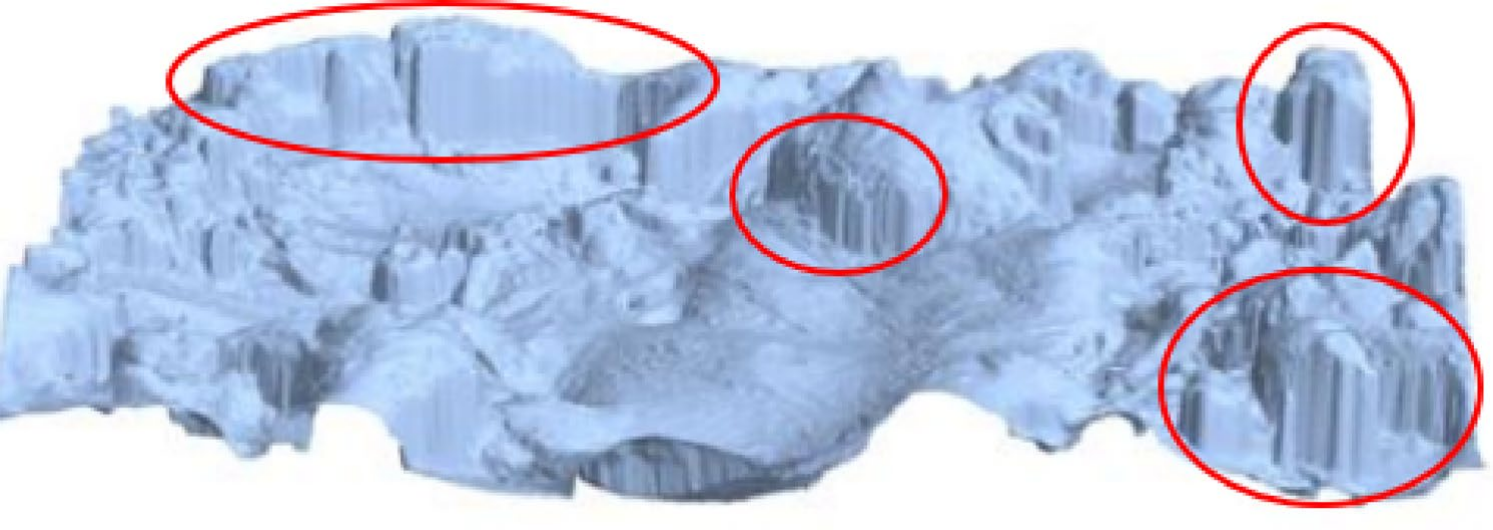
View of the surface after EDM machining with re-entrant features, with marked smoothings and geometry simplifications of the surface fragments with re-entrant features^50^.

Conventional measurement methods struggle to capture the complex geometry of surfaces with re-entrant features. These techniques often oversimplify concave regions, as data are collected mainly from surface peaks, leading to flattened representations. As shown in Fig. 6, curved and intricate geometries appear smoothed and simplified.

**Fig. 7:**
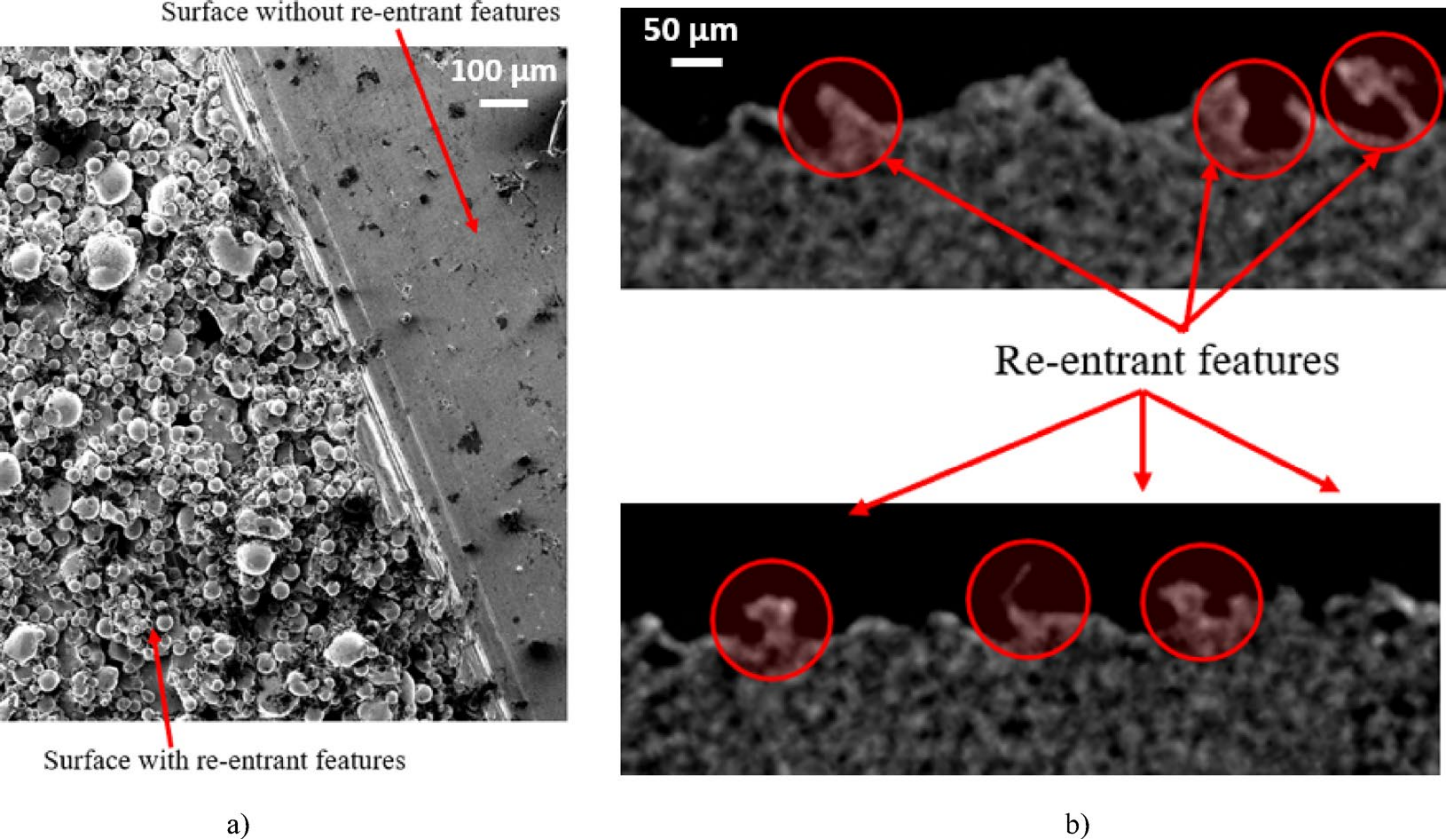
(**a**) comparison of the surface after PBF printing with and without re-entrant features; (**b**) view of re-entrant features of the tested samples – 2D view.

µCT measurement is the only method that allows full determination of surface occurrence and geometry after PBF (Powder Bed Fusion), enabling identification of re-entrant features for further analysis. Other surface measurement techniques only capture the upper parts of re-entrant features, missing the underlying spaces and potentially distorting results. Figure 7a) shows two PBF-fabricated surfaces—one with re-entrant features and one turned, without re-entrant geometry—measured via Scanning Electron Microscope. The left surface’s spherical geometry prevents observation beneath larger powder sinterings. Figure 7b) shows 2D projections from MJF tomographic images, where re-entrant surfaces are also present and fully analyzable. Analysis of tomographic images showed that the sizes of re-entrant structures ranged from 10 μm to 60 μm, with these values determined based on the diameter of spheres representing their geometry.

**Fig. 8:**
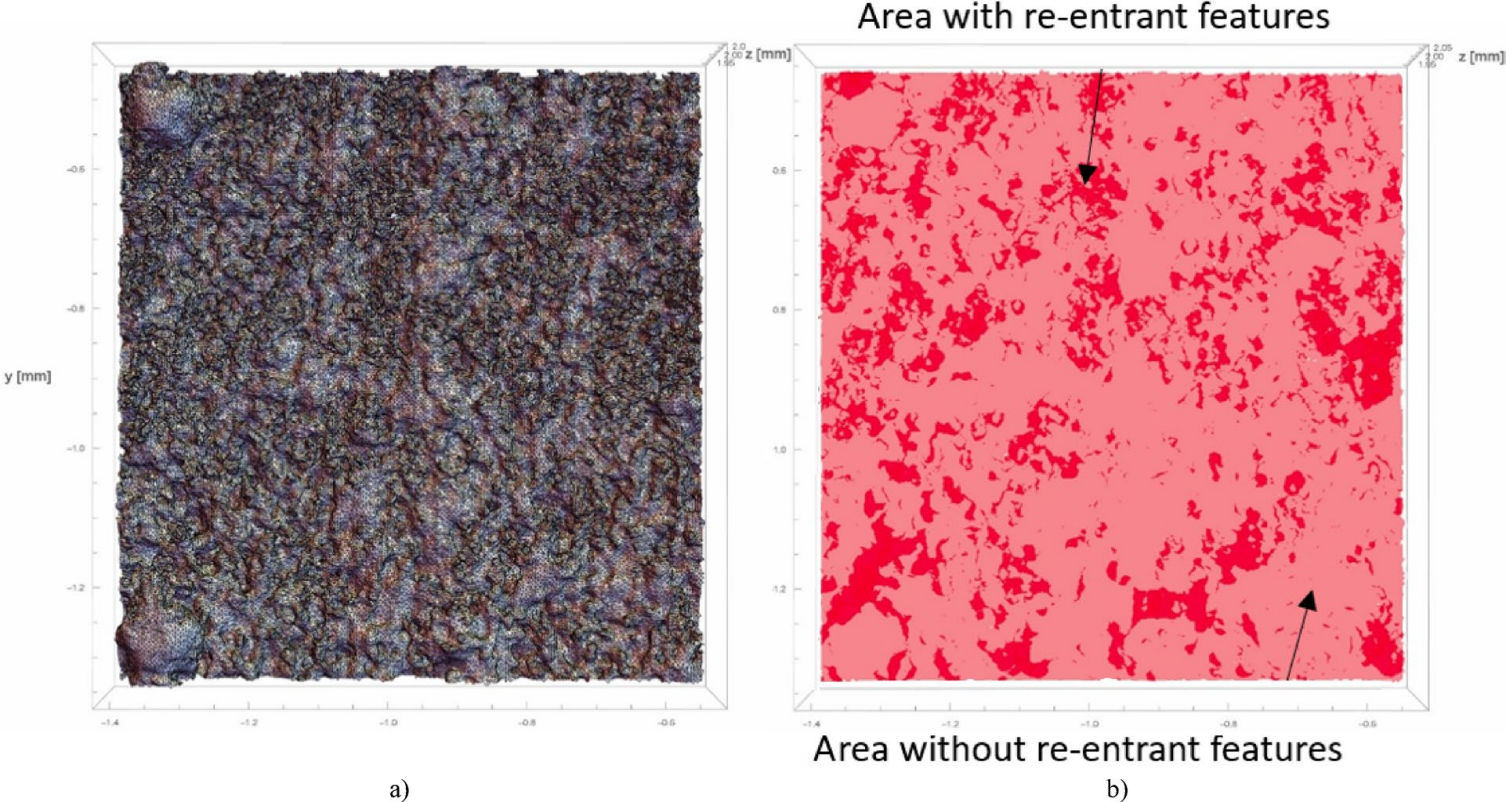
Surface with re-entrant features: (**a**) view of the extracted surface model; (**b**) projected (**a**) surface onto the reference plane - darker areas are the zone of re-entrant features, lighter areas without re-entrant features.

Consequently, following tomographic measurements, a decision was made to quantitatively ascertain the occurrence of re-entrant features within the entire analyzed surface. An algorithm was devised to project the overall surface onto a reference plane, associated with the surface form, which is perpendicular to the z-axis. Due to that assumption, projection can be done by reducing z-component of every triangle vertex. The resulting set is composed of triangles which vertices have only two components, i.e. *x* and *y*. This method uses overlapping projected areas to quantify re-entrant feature prevalence across the analyzed surface. Figure 8b shows the projection onto a reference plane, with darker regions indicating re-entrants and brighter areas representing conventional surfaces, where each abscissa coordinate corresponds to an ordinate coordinate. Figure 8a presents the extracted surface model used for quantitative analysis of re-entrant features.

To establish a specific reference in the analysis of re-entrant features, it was decided to define them using two quantitative parameters. These parameters are as follows:

- *r*_*1*_ – represents the surface area of re-entrant features projected onto the nominal surface. It indicates the percentage of the actual re-entrant surface area compared to the nominal surface area, which was assumed to be 100%. Therefore, if the result of parameter *r*_*1*_ exceeds 100%, it means that the measured re-entrant surface area is larger by a certain percentage (exceeding 100%) than the reference surface area. For instance, if the *r*_*1*_ parameter is 150%, it implies that the measured re-entrant surface area is 150% of the reference area – 50% larger.
  $${r_1}=\frac{{{A_{{\text{re-entrant}}}}}}{{{A_{{\text{nominal}}}}}} \times 100\%$$
  where:
  *A*_re−entrant_ – measured surface area of the re-entrant features *A*_nominal_ – reference (nominal) surface area, assumed to be 100%.
- *r*_*2*_ – signifies the proportion of the nominal surface area occupied by re-entrant features. It was defined that 0% represents surfaces without re-entrants, while 100% represents surfaces solely comprised of re-entrants. This indicates the proportion of the projected area onto the reference plane that constitutes the surface distinguished by re-entrant features. Referring to Fig. 8, it can be deduced that this percentage corresponds to the dark red area relative to the entire reference plane. For example, if the *r*_*2*_ parameter is 40%, then 40% of the entire reference surface area is occupied by the projected surface with re-entrant features.
  $${r_2}=\frac{{{A_{{\text{re-entrant}}\;{\text{proj}}}}}}{{{A_{{\text{nominal}}}}}} \times 100\%$$
  where:
  *A*_re−entrant proj_ – re-entrant surface area projected onto the reference plane.

Considering re-entrant surfaces is essential for a thorough surface assessment as they can influence various factors related to contact mechanics and surface properties. Yamaguchi^51^ explored the impact of surfaces with re-entrant features on contact with food products, focusing on soy sauce and rapeseed oil. The author demonstrated significant hydrophobic and oleophobic properties of the created surfaces. Conversely, Sow et al.^52^ investigated surface self-cleaning and corrosion resistance. They confirmed the self-cleaning properties of coatings with re-entrant features using three types of contaminant particles: fine graphite dust, table salt, and aluminum powder. Additionally, the surfaces exhibited corrosion resistance in a 3.5% NaCl solution, with a very low corrosion rate of 0.328 nm/year. Lee et al.^53^ demonstrated that wetting and flow parameters at the top of re-entrant geometry exhibited anisotropic properties. Meanwhile, Houngkamhang et al.^54^ examined bacterial distribution on the surface. They observed that microstructures with re-entrant characteristics prevented bacterial adhesion, which could significantly contribute to the potential use of such surfaces for antibacterial purposes in healthcare.

### Post-processing

The sandblasting was employed for post-processing. As outlined in Sect. 1, this technique has minimal impact on the resulting surface. Sodium-silica glass beads (free of free silica) served as the abrasive material. This substance enables controlled cleaning without inducing macroscopic or structural damage to the part, while still allowing for surface modification, which is further characterized in this study. Additionally, it is effective for removing burrs and satinizing metal parts used in aluminum, brass, or stainless steel applications. Chemically inert and insoluble in water and oil, it guarantees no contamination of the processed parts. Table 5. detail the physico-chemical properties of the abrasive utilized^55^.

In the chosen post-processing method, both the pressure of the abrasive spray and the size of the abrasive material play pivotal roles. While the chemical composition holds less significance, maintaining a balance between the abrasive material and the workpiece is imperative. The sizing of the abrasive material must align with the dimensions of the powder used for MJF. Given that MJF powder typically boasts a diameter of approximately 60 μm, it is crucial for the abrasive to possess a diameter no smaller than that of the powder. Should the abrasive be undersized compared to the powder, there exists a risk of it infiltrating the surface geometry, embedding within its structure. This scenario is particularly pronounced for surfaces featuring re-entrant attributes, where the abrasive might linger beneath these features, skewing subsequent surface measurements, focusing on the abrasive’s geometry rather than that of the MJF element’s surface.

An analysis of the abrasive material was also performed using µCT. The measurement parameters were defined as follows The voxel size stood at 0.898 μm, with a voltage of 130 kV, a current of 90 µA, an exposure time of 300 ms, and a projection count of 2000.

**Table 5:** Physical properties of the abrasive used.

Sandblasting beads physical properties							
	Beads size [µm]	Softening point [°C]	Density (kg/L)	Mohs hardness	Form		
Sodium-silica glass	75–150	730	1,5 − 1,6	6	Smooth, translucent spheres		

Sandblasting beads chemical composition							
	SiO_2_ [%]	Na_2_O[%]	CaO[%]	MgO [%]	Al_2_O_3_ [%]	K_2_O [%]	Fe_2_O_3_ [%]
Sodium-silica glass	70–75	12–15	7–12	< 5	< 2,5	< 1,5	< 0,5

In order to minimize the impact of the operator and the impact resulting from the sandblasting process itself on the resulting surface, it was decided to clean and subsequently analyse 30 surfaces. A Renfert Basic quattro IS micro-sandblasting machine was used here. The abrasive jet pressure was 4 bar.

### Chemical properties

Despite applying optimal bead diameters for sandblasting (Sect. 4.1), it was necessary to ensure that no abrasive residues remained on the sample surfaces, as residual glass beads could distort post-processing results by masking the actual surface geometry. To verify surface cleanliness, a chemical analysis of randomly selected samples was performed using a JEOL SEM-FEG 7100 F microscope equipped with EDS X-act and EBSD Nordlys systems. The analysis, based on energy-dispersive X-ray spectroscopy (EDS), enabled detection of elements characteristic of the abrasive but absent in PA12. Their presence would indicate contamination, while their absence confirmed clean sample surfaces. The technical parameters of the applied equipment are listed in Table 6.

**Table 6:** SEM paremeters.

SEM machine characterisation	
Microscopy	JEOL SEM-FEG model 7100 F
Resolution	2 nm at 1 kV, 1.6 nm at 5 kV, 1.2 nm at 15 kV
Probe current	1pA – 600 nA
Detectors	EDS X, EBSD

## Results

### Post-processing

If the abrasive diameter significantly surpasses that of the powder, glass beads may struggle to comprehensively cleanse the surface. Consequently, the abrasive material might fail to reach all surface geometries, leaving unmelted powder residues on the element’s surface. Hence, it’s paramount to opt for abrasive material slightly larger than the powder’s diameter, ensuring thorough surface cleansing while preventing abrasive residues from lingering within the cleaned element structures. Consequently, the decision was made to select the diameter of the glass beads in accordance with Table 5.

**Fig. 9:**
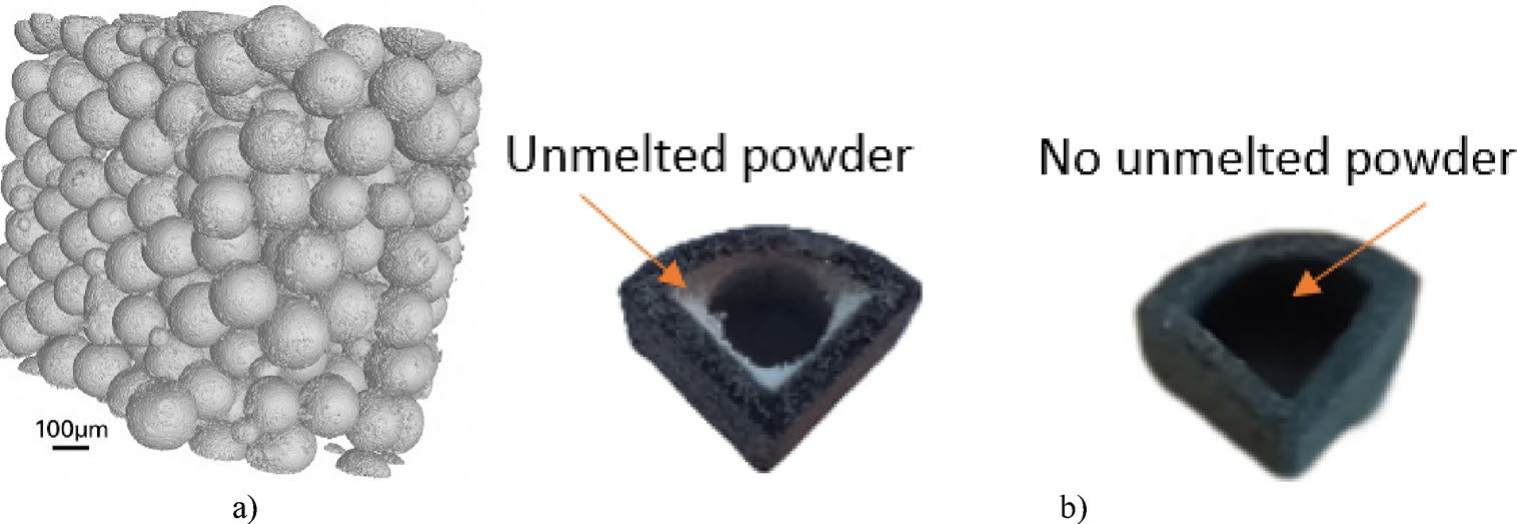
View of: (**a**) the model of the measured glass beads from the tomographic analysis; (**b**) the uncleaned and cleaned inner surface of the measurement sample.

Given the significance of subsequent procedures, final outcomes, and surface evaluations following post-processing before sandblasting, it was deemed necessary to measure the diameter of the glass beads utilized in the process. This step was vital to validate the procedure’s accuracy and to forestall any residual abrasive material from persisting within the measurement element structures. For this measurement, a computer tomograph, as described in Sect. 3.2.1, was employed. Leveraging computer tomography enabled the comprehensive portrayal of the glass beads’ geometry from all angles (360°), facilitating precise alignment of the surrogate measurement elements with the overall geometry of the glass beads. In contrast, other measurement methodologies typically permit observation of the glass beads’ geometry from a singular perspective, directly influencing measurement quality and reliability. The model view of the measured glass beads from tomographic analysis is depicted in Fig. 9a.

**Fig. 10:**
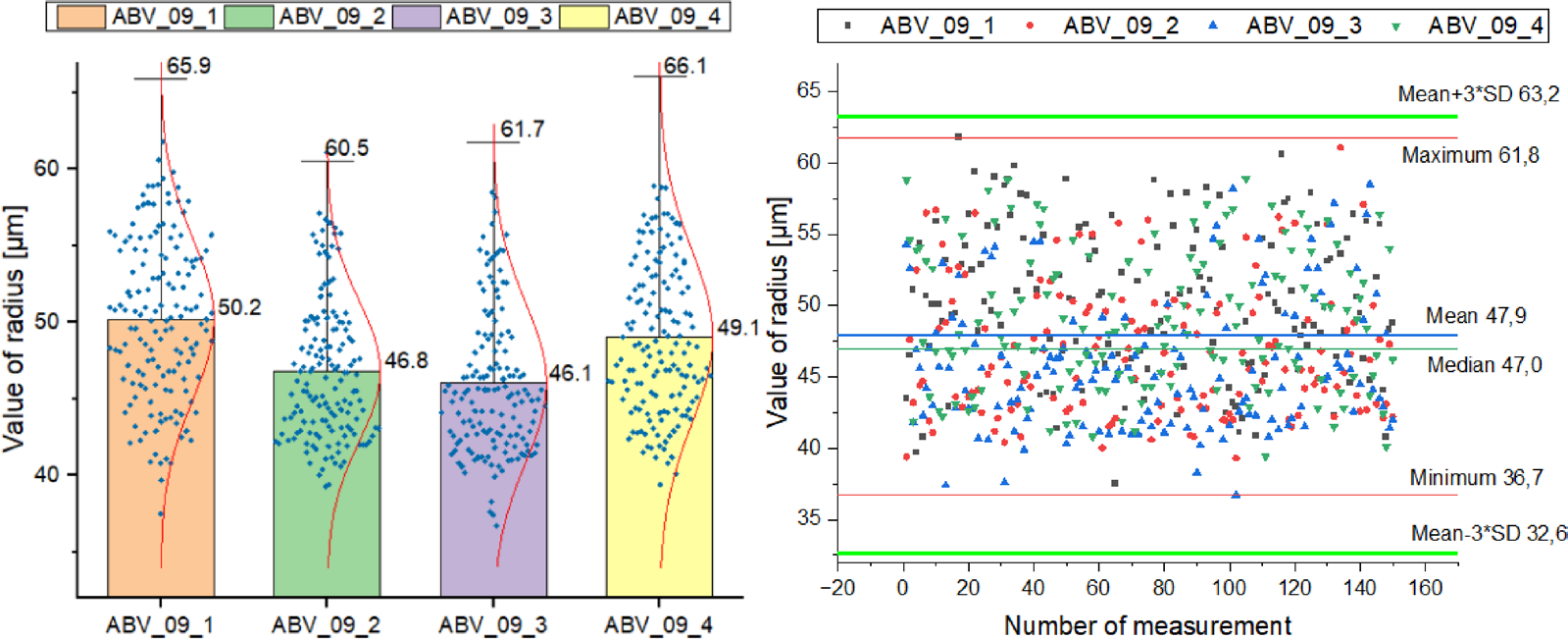
(**a**) A graph showing the results obtained for each series of measurements of the abrasive used; (**b**) a graph showing a summary of the measurement results for all measurement series; The “Bar” section of the chart displays the average value of the results, while the “Whisker” section represents the average value plus 3 standard deviations, in line with the three-sigma rule (where 99.7% of feature values fall within ≤ 3σ from the expected value), with the population count distributed across one-standard-deviation-wide intervals. The red curve depicts the probability density of the normal distribution.

According to the manufacturer’s documentation, the bead radius should range between 37.5 μm and 75 μm. To validate the glass bead size, 600 measurements were conducted and divided into 4 series, each comprising 150 measurements of the abrasive bead radius. The results for each series are depicted in Fig. 10a. A Bar & Whisker chart was generated for each series, with blue dots indicating the position of each measurement within the series. Each consecutive series is represented separately (ABV – type of shot blaster; 0898 – voxel size; 1–4 – series number) (Table 7):

**Table 7:** Abrasive size results for individual measurement series.

Standard	Average [µm]	Average + 3σ [µm]
ABV_09_1	50.2	65.9
ABV_09_2	46.8	60.5
ABV_09_3	46.1	61.7
ABV_09_4	49.1	66.1

The fluctuations in the mean value are within 4 μm, without exceeding the manufacturer’s declared values. Analyzing the above data, it can be concluded that the measurements were correctly executed due to the achieved normal distribution and not exceeding the average plus 3σ for each series. The obtained normal distribution of cumulative distribution functions confirms this alignment with the manufacturer’s declarations.

Figure 10b illustrates the distribution of all measurements across the 4 series. Similarly, the values of the average plus 3σ and additionally − 3σ are marked. All measurement values fall within the assumed range. The average of all measurements was 47.9 μm, with a median of 47.0 μm. The maximum measurement value was 61.8 μm and the minimum was 36.7 μm. The obtained average value falls at the lower end of the abrasive manufacturer’s permissible values range. This suggests that glass beads will effectively access all surface geometries. One measurement fell outside the manufacturer’s measurement range - deviating by 0.8 μm from the lower range. However, since this was an isolated measurement within acceptable limits, it’s considered a measurement error. Analyzing the individual data, it can be concluded that all results fell within the abrasive size range declared by the manufacturer. The average value, falling within the lower range of permissible values, ensures effective and appropriate surface cleaning (considering the MJF powder size: 60 μm). The measurements validate the manufacturer’s assumptions.

Once the abrasive size analysis was completed, it was possible to proceed to cleaning the samples. A view of the uncleaned and cleaned inner surface of the sample can be seen in Fig. 9b.

### Surface topography

#### Analysis of the occurrence of re-entrant surfaces before post-processing

**Fig. 11:**
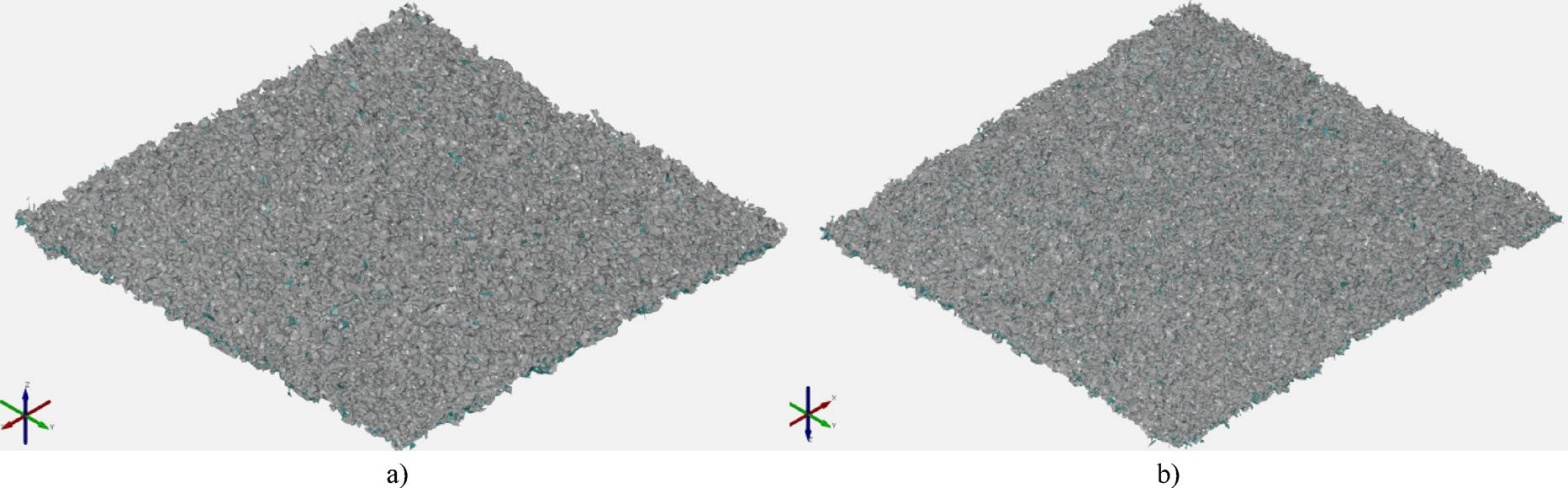
View of samples surfaces before post-processing - µCT measurement: (**a**) 1. surface – sample 8: 3 × 3 mm; (**b**) 2. surface – sample 13: 3 × 3 mm *a) b)*

After performing tomographic measurements, it was necessary to export individual measured surfaces for further analysis. The surfaces were defined in STL format. The models of exported surfaces are presented in Fig. 11.

**Fig. 12:**
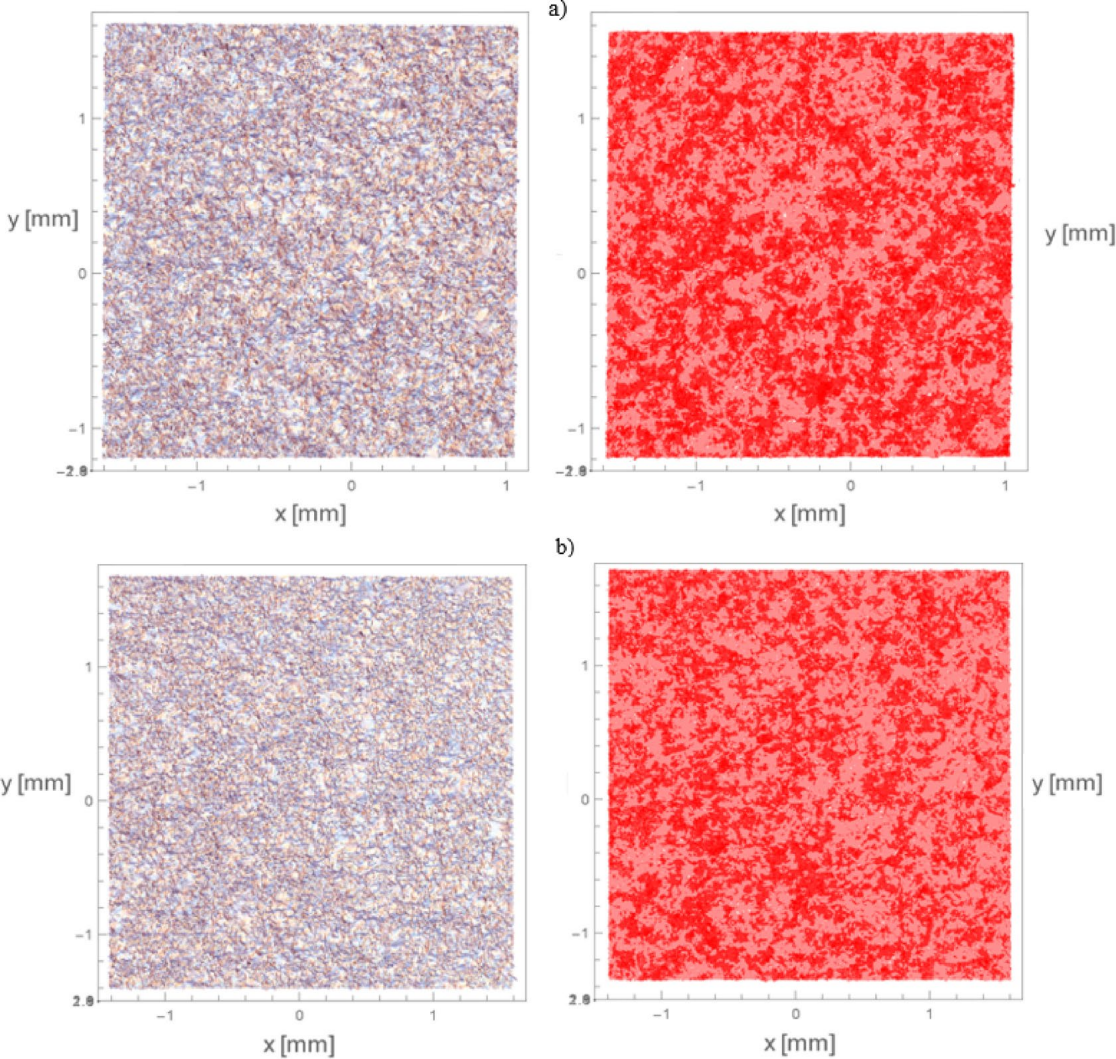
Top view of the measured surfaces: on the left before the projection on the right after the projection: (**a**) Surface 2 for sample 22 *r*_*1*_ = 259.04% *r*_*2*_ = 56.43%; (**b**) Surface 1 for sample 22 *r*_*1*_ = 229.91% *r*_*2*_ = 48.34%.

In accordance with Chap. 3.2.3., surfaces following MJF processing exhibit re-entrant features. Consequently, the investigation aimed to ascertain the alterations in these features post sandblasting. Initially, the generated surfaces pre post-processing underwent quantitative analysis of the present re-entrant geometries. Subsequently, the acquired surfaces were projected onto a flat reference plane to visualize the occurrence of re-entrants along the normal axis to the reference plane. Figure 12. illustrates the top view of the measured surface both pre and post projection. Regions highlighted in dark red indicate areas where re-entrant features are present, while those in light red denote regions devoid of such features.

**Fig. 13:**
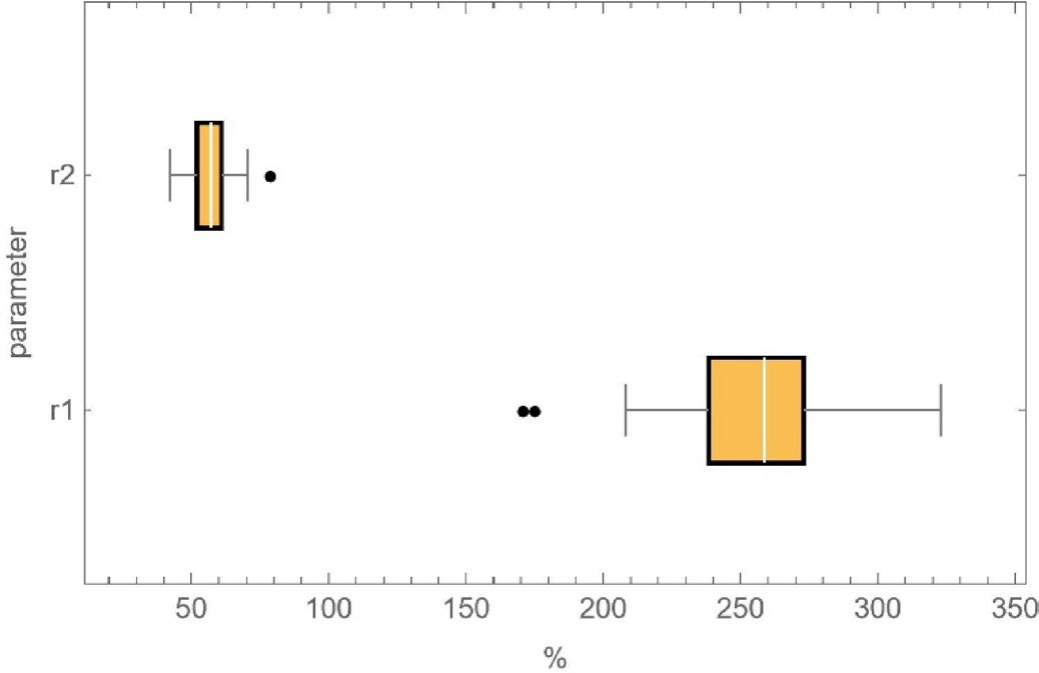
Graph of the distribution of the magnitude of the parameter *r*_*1*_ and *r*_*2*_ with centile interpretation (before sandblasting); For both parameters, the “Whisker” section indicates the maximum and minimum values of the analysis results. The “Bar” section highlights the highest concentration of results (between the 25th and 75th percentiles). Additionally, average values are denoted on the “Bar” section with a white line.

Figure 13 presents a comparison of all analyzed surfaces. It illustrates the same distribution of parameters *r*_*1*_ and *r*_*2*_. Furthermore, ranges marked with points for both parameters are noted. These indicate results that deviate from the overall dataset and are outside the scope of the analysis – they were not included in the interpretation of results and are considered outliers. Based on the information above, it can be observed that the mean values are close to 260% for parameter *r*_*1*_ and 60% for *r*_*2*_.

#### Analysis of the occurrence of re-entrant surfaces after post-processing

**Fig. 14:**
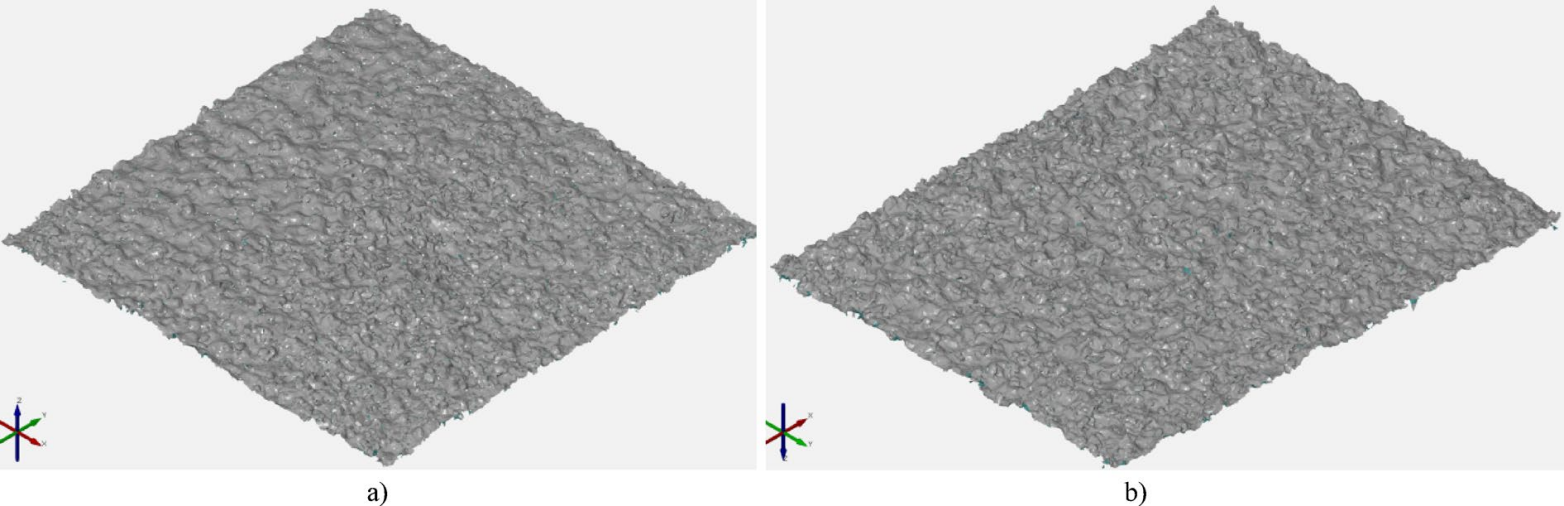
View of samples surfaces after post-processing - µCT measurement: (**a**) 1. surface – sample 8 3 × 3 mm; (**b**) 2. surface – sample 13 3 × 3 mm.

Again after performing tomographic measurements, it was necessary to export individual measured surfaces for further analysis. The surfaces were defined in STL format. The models of exported surfaces are presented in Fig. 14.

**Fig. 15:**
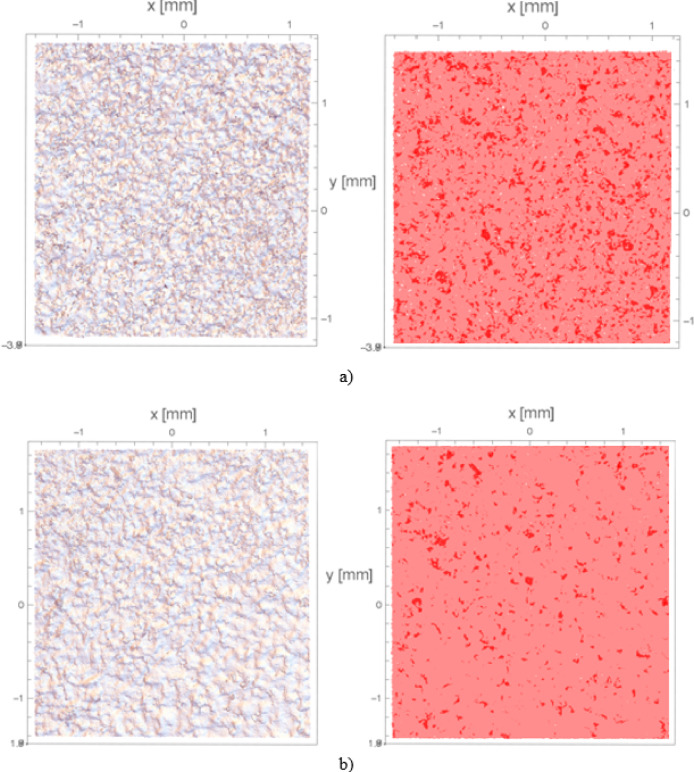
Top view of the measured surfaces: on the left before the projection on the right after the projection: (**a**) Surface 2 for sample 22 *r*_*1*_ = 143.32% *r*_*2*_ = 15.70%; (**b**) Surface 1 for sample 22 *r*_*1*_ = 120.60% *r*_*2*_ = 5.60%.

The extracted samples underwent analysis for the presence of re-entrant features. The procedure of the study was conducted identically to that in Chap. 4.2.1. Figure 15 illustrates the top view of the measured surface both pre and post projection. Regions highlighted in dark red indicate areas where re-entrant features are present, while those in light red denote regions devoid of such features.

**Fig. 16:**
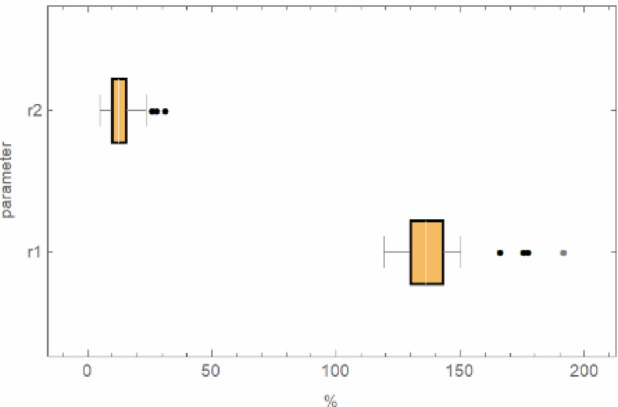
Graph of the distribution of the magnitude of the parameter *r*_*1*_ and *r*_*2*_ with centile interpretation (after sandblasting*);* For both parameters, the “Whisker” section indicates the maximum and minimum values of the analysis results. The “Bar” section highlights the highest concentration of results (between the 25th and 75th percentiles).

Figure 16 presents a comparison of all analyzed surfaces after post-processing. It illustrates the same distribution of parameters *r*_*1*_ and *r*_*2*_. Average values are denoted on the “Bar” section with a white line. Furthermore, ranges marked with points for both parameters are noted. These indicate results that deviate from the overall dataset and are outside the scope of the analysis – they were not included in the interpretation of results and are considered outliers. Based on the information above, it can be observed that the mean values are close to 135% for parameter *r*_*1*_ and 13% for *r*_*2*_.

Comparing the results before and after the post-processing operation, it is evident that the areas where re-entrant features occurred were reduced almost fivefold, considering parameter *r*_*2*_. However, the total area occupied by re-entrant features was almost halved, with reference to parameter *r*_*1*_. The average value of parameter *r*_*2*_ before sandblasting was 60%, whereas after, it decreased to 13%. Similarly, the average value of parameter *r*_*1*_ before sandblasting was 260%, and after, it reduced to 135%.

These findings suggest that sandblasting, as part of the sample cleaning process to remove unbound powder, significantly affects the surface characteristics, leading to a reduction in the occurrence of re-entrant features. This could have a significant impact on the practical applications of MJF printed elements, emphasizing the importance of appropriate post-processing operations to achieve the desired surface properties for specific uses.

#### Surface analysis - multiscale geometric surface characterizations

The characterization of surfaces before and after post-processing is complemented by multiscale geometric analyses, described here with the parameters of the relative area (*Srel*) and area-scale complexity (*Asfc*). The surfaces produced additively in Multi Jet Fusion technology, analyzed in these studies, are described by isotropic texture. Area-scale analyses better characterize such surfaces than length-scale analyses, which are more dedicated to anisotropic surfaces that allow for showing relative lengths and complexity depending on the directionality of geometric surface features. Isotropic textures can be reliably analyzed in an area-scale approach.

**Fig. 17:**
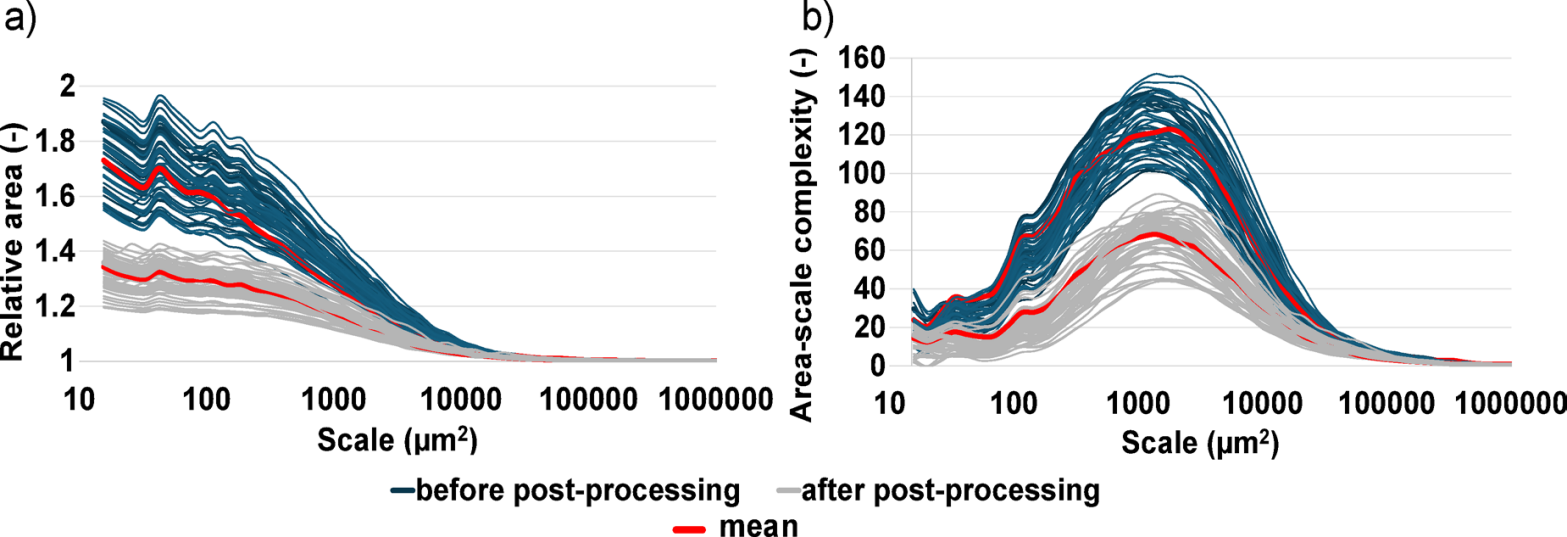
Area-scale plots: (**a**) relative areas versus scale for the surfaces before and after post-processing, (**b**) area-scale complexity versus scale for the surfaces before and after post-processing. Note: The plot shows 60 surfaces with measured relative areas before post-processing and 60 surfaces after post-processing.

For surfaces before and after post-processing, it was possible to calculate relative areas at multiple areal scales. At fine scales, a clear discrimination between these two groups of surfaces is visible. Post-processed surfaces describe smaller relative areas, related to smoothing the texture. In addition, based on 60 measurements of different post-processed surfaces, a smaller fluctuation in relative areas can be observed than for surfaces before post-processing. Despite maintaining the same additive manufacturing process parameters, the created surfaces are characterized by certain fluctuations in topographic heights, which are narrowed after post-processing. As the scale of observation increases, the relative areas of the surfaces decrease. For coarser scales above 10,000 µm^2^, the relative areas before and after post-processing start to equalize. Area-scale complexity is another multiscale geometric parameter enabling the characterization and discrimination of surfaces before and after post-processing. Similarly to relative areas, complexity is higher for surfaces before post-processing with a greater share of re-entrant features. *Asfc*, similar to *Srel*, levels off for coarser scales. At finer scales, the discrimination is visible, especially at range of scales from 100 µm^2^ to 10,000 µm^2^. The area-scale complexity of post-processed surfaces is about two times smaller compared to pre-post-processed surfaces at the best scale to observe this discrimination of 1000 µm^2^. Mean relative area and mean area-scale complexity calculated from 120 (60 before sandblasting, 60 after sandblasting) measurements show a clear difference for the surfaces before and after post-processing (Fig. 17). The difference between them is not visible for coarser scales.

**Fig. 18:**
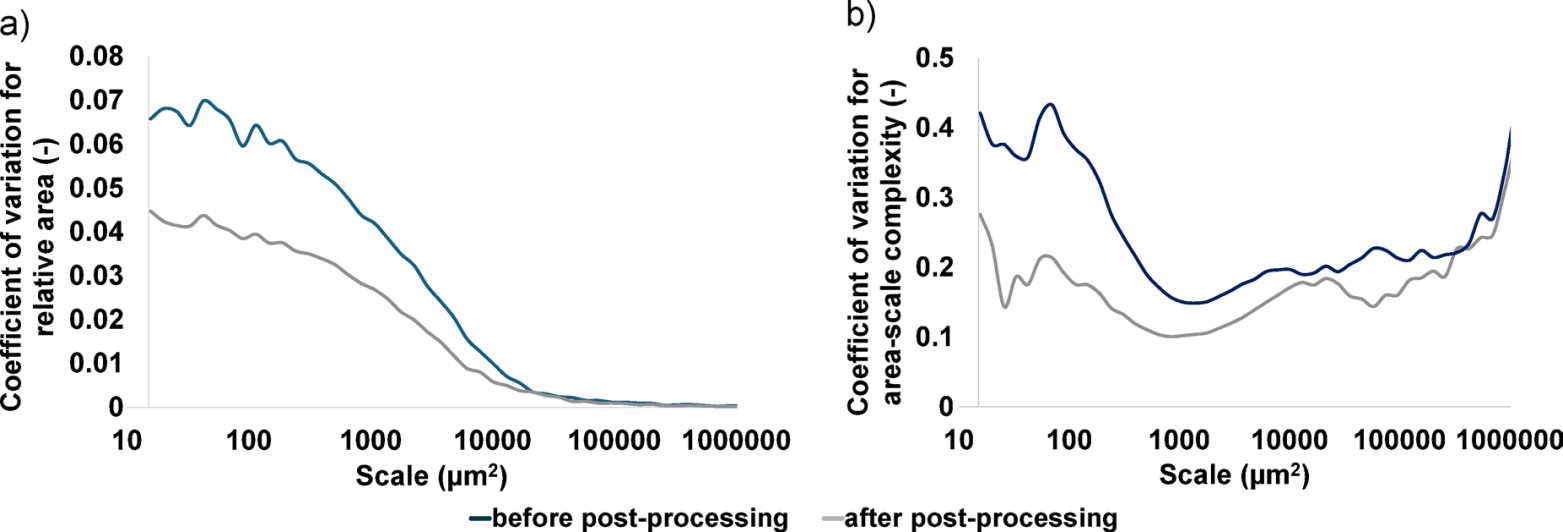
Coefficient of variation versus scale for the surfaces before and after post-processing: (**a**) relative area, (**b**) area-scale complexity. The mean and coefficient of variation are calculated based on 60 surfaces before post-processing and 60 surfaces after post-processing.

Repeated topographic measurements generate some fluctuation in the results (Fig. 18)^56^. The coefficient of variation is larger for the surfaces before post-processing, both for relative area and complexity at finer scales. Surfaces with re-entrant features are therefore characterized by greater non-repeatability in relative areas and complexities at finer scales. Sandblasting allows smoothing these surfaces and reducing the proportion of re-entrant features, which leads to lower coefficients of variance between repeated measurements. Post-processed surfaces are more repeatable.

**Fig. 19:**
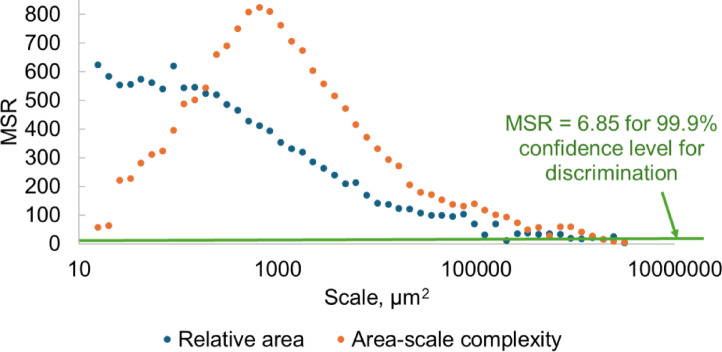
Mean square ratios of relative area and area-scale complexity for discriminating the surfaces before and after post-processing. horizontal line indicate the minimum mean square ratio (6.85) for 99.9% confidence level for discrimination.

To determine the statistical significance of the discrimination between surfaces before and after post-processing, univariant ANOVA was used and mean square ratios (MSRs) were calculated (Fig. 19). The critical MSR value of 6.85 sets the surface discrimination barrier. At the 99.9% confidence level, relative areas and area-scale complexities allow for the discrimination of surfaces before and after postprocessing for almost the full range of scales, excluding very coarse scales, for which all surfaces are statistically similar.

#### Surface analysis – ISO parameters

**Fig. 20:**
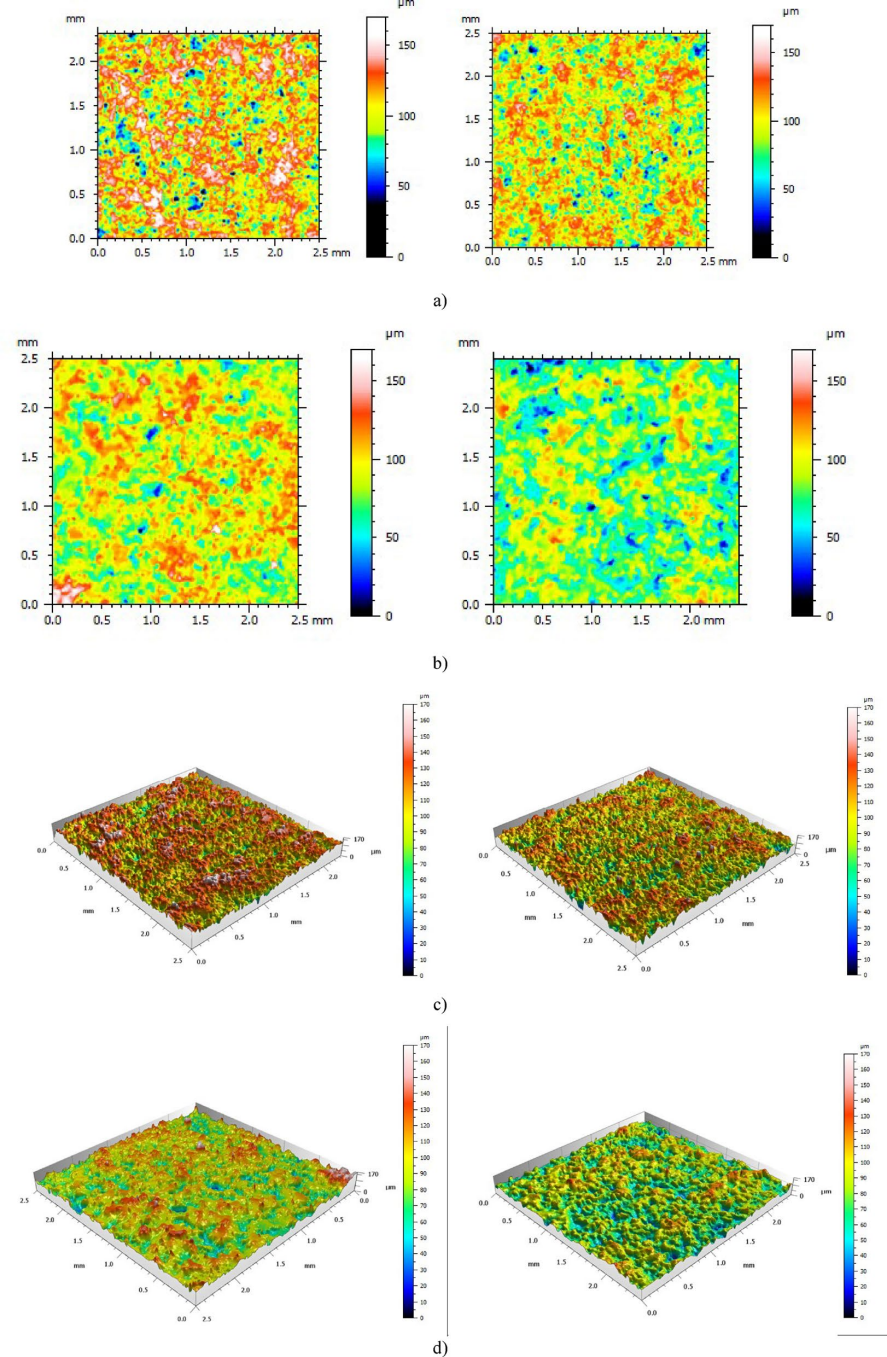
Topographic views of the measured surfaces – left before, right after post-processing: (**a**) 2D view: sample 19 surf. 1; (**b**) 2D view: sample 28 surf. 2; (**c**) 3D view: sample 19 surf. 1; (**d**) 2D view: sample 28 surf. 2.

Figure 20 presents 2D and 3D topographic views of selected samples: sample 19 (surface 1) and sample 28 (surface 2). In the presented examples, a significant change in the surface characteristics after post-processing can be observed when compared to the original surfaces. Notably, there is a marked reduction in peak heights, which led to a flattening of the surface. Additionally, it can be seen that sandblasting resulted in considerable surface homogenization. The geometric nature of the surface became more organized and uniform. The height of the peaks was reduced, and the deep penetration of the abrasive medium led to smoothing of the depressions. These topographic observations corroborate the indications and relationships derived from roughness parameter analysis as well as multiscale analysis. The topographic views clearly show that the surface geometry underwent significant changes, which must be considered in the context of manufacturing and technology. Furthermore, it is worth noting that the surfaces after sandblasting still differ in the specific features of individual peaks and valleys. This results from the inherent nature of abrasive blasting, which does not allow for the creation of identical surfaces but only ones that are similar. These differences are further influenced by the varying degree of disorder and the initial dimensions of surface recesses and elevations prior to post-processing. This indicates that surfaces of samples produced under identical printing parameters can still vary significantly. Therefore, it is important to highlight that despite differences in the initial surface characteristics, abrasive treatment enabled effective smoothing and homogenization of the surfaces.

**Fig. 21:**
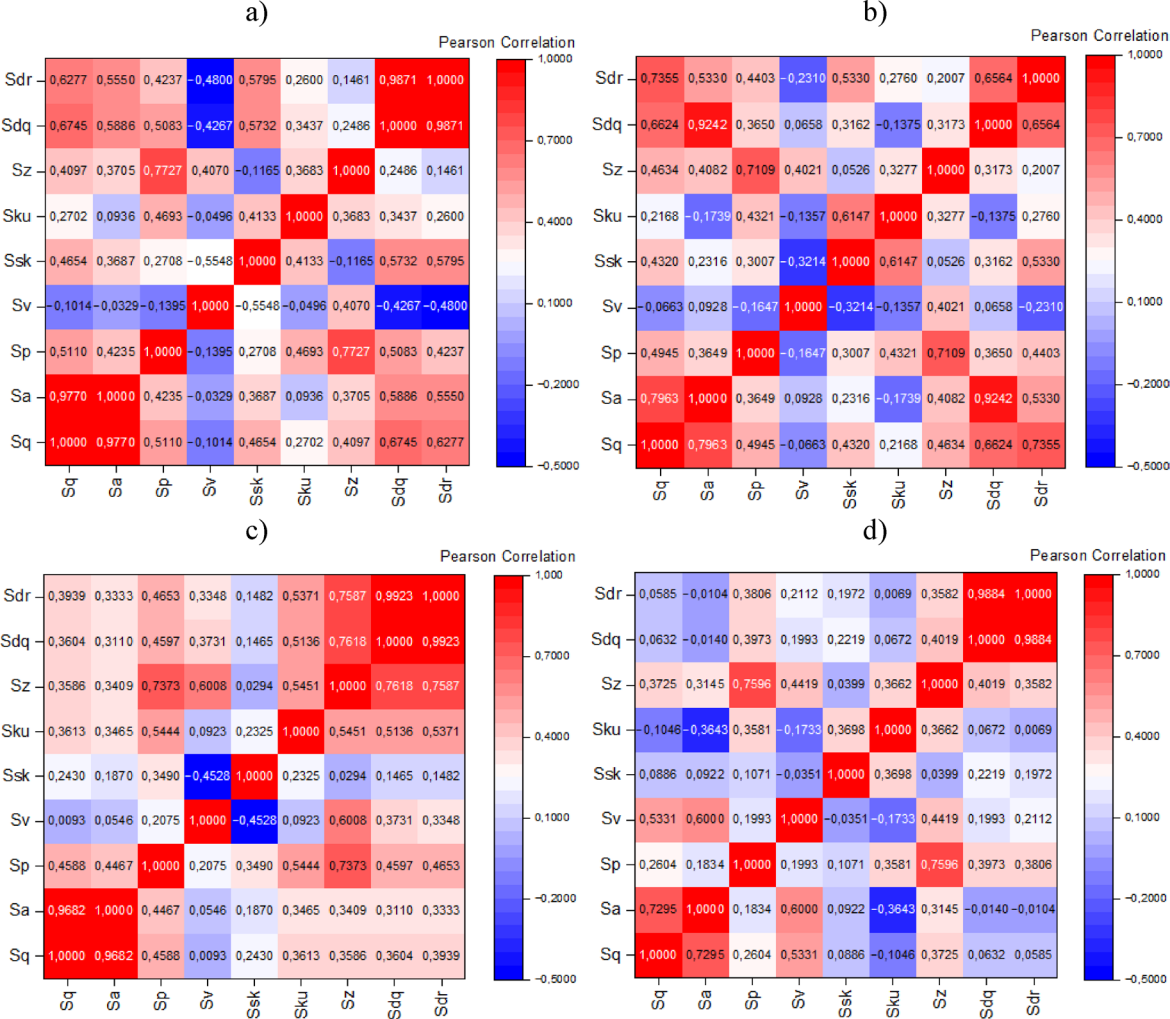
Correlation matrices of selected surface roughness parameters for the measured samples: (**a**) surface 1 before sandblasting; (**b**) surface 2 before sandblasting; (**c**) surface 1 after sandblasting; (**d**) surface 2 after sandblasting.

In this study, surface roughness parameters were calculated and organized based on their group affiliation or the strength of their mutual correlation. To present the data as clearly as possible while maintaining readability, correlation matrices were used, as shown in Fig. 21. The strongest correlations were observed between the parameters *Sa* and *Sq*, as well as *Sdq* and *Sdr*. *Sa* accounts for all height deviations from the reference plane, while *Sq* is more sensitive to larger irregularities. A strong correlation (r close to 1) between these two parameters was observed after sandblasting for surface 1. Surface 2 showed a slightly weaker correlation (though still within the range of high significance) both before and after sandblasting, which may be related to the orientation and positioning of the sample during forming.

High correlation values (*r* ~ 1) across all cases indicate a uniform distribution of surface roughness without sharp or abrupt defects. This suggests that the roughness distribution is symmetrical and homogeneous an increase in average height values (*Sa*) is accompanied by an increase in their variability (*Sq*). This is expected behavior and confirms the quality of the measurements and the stability of the surface topography before and after sandblasting.

Strong correlations between *Sdq* and *Sdr* were also recorded both before and after sandblasting. However, an increase in Pearson correlation was observed after sandblasting, especially for surface 2, reaching *r* ~ 1. The relationship between *Sdq* and *Sdr* remained consistent samples with higher slope values (*Sdq*) continued to exhibit a higher degree of surface development (*Sdr*). Furthermore, as indicated by the correlations, the strong post-treatment correlation between *Sdq* and *Sdr* confirms that these parameters jointly describe the variability of surface topography effectively. Before sandblasting, *Sdq* and Sdr were already strongly correlated, suggesting that higher local slopes were closely associated with more developed surfaces. After sandblasting, this relationship became even stronger, possibly indicating that, after smoothing, changes in slope increasingly determine structural variability. The increase in Pearson correlation after treatment further confirms the homogenization of the surface. The high correlation between these parameters points to the geometric consistency of the topography and indicates that the microstructure (gradients) strongly influences the macroscopically developed surface.

**Fig. 22:**
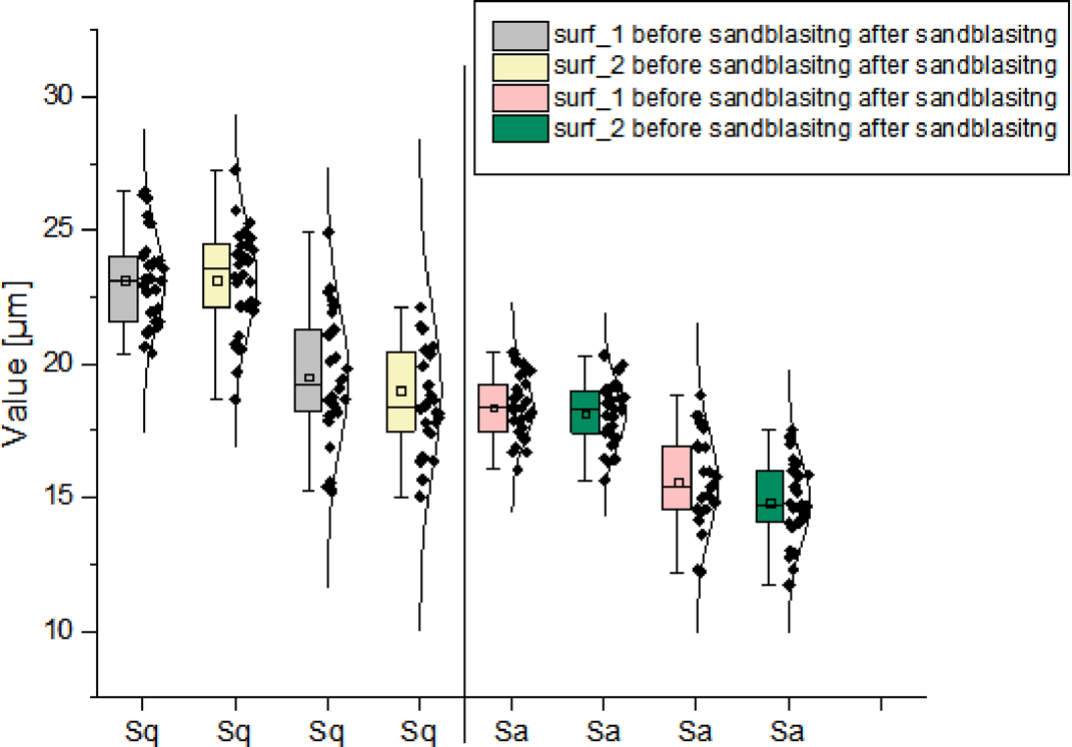
Distributions of the values of the parameters *Sa* and *Sq*: (**a**) including the distribution of quartiles (**b**) including the mean values and standard deviation.

Figure 22 presents the mutual distribution of the *Sa* and *Sq* parameters for the two analyzed surfaces, based on 30 samples measured before and after sandblasting. For each case, the mean and median values were indicated, considering all samples each with surface 1 and surface 2. According to Fig. 22, the box and whisker plot elements represent the interquartile range (IQR) the difference between the third quartile (Q3, 75th percentile) and the first quartile (Q1, 25th percentile). The whiskers mark the minimum and maximum values that do not exceed 1.5 × IQR. Additionally, a point distribution of individual sample values was included along with their respective distributions. The near-normal shape of the distributions, the inclusion of results within appropriate quartile ranges, and the clustering of values all confirm the reliability of the data. From the plots, it can be concluded that *Sa* and *Sq* values for both surfaces before and after sandblasting are at a comparable level. Values for surface 1 are slightly higher, but the relative relationship remained unchanged after sandblasting. After treatment, the *Sq* values fell within the range of *Sa* values prior to sandblasting, meaning their magnitude was reduced to the level of pre-sandblasting *Sa*. Additionally, the *Sa* value after sandblasting was significantly reduced to around 15 μm (-the initial value was 20 μm). These results indicate that the surfaces became smoother and surface irregularities were diminished. Although sandblasting could initially be expected to increase surface roughness, in practice, it removed sharp peaks and valleys, smoothing out the microstructure, which was originally characterized by high irregularity due to the presence of re-entrant features. A micro-peeling process occurred, where peaks and valleys were leveled out. The surface became more uniform, with regularized roughness. The reduction in mean and standard deviation of *Sa* and *Sq* suggests that extreme surface irregularities were diminished. Furthermore, the longer bars for the pre-sandblasting samples indicate greater variability, while the shorter bars after sandblasting point to more homogeneous data, demonstrating that the sandblasting process stabilized the surface topography.

**Fig. 23:**
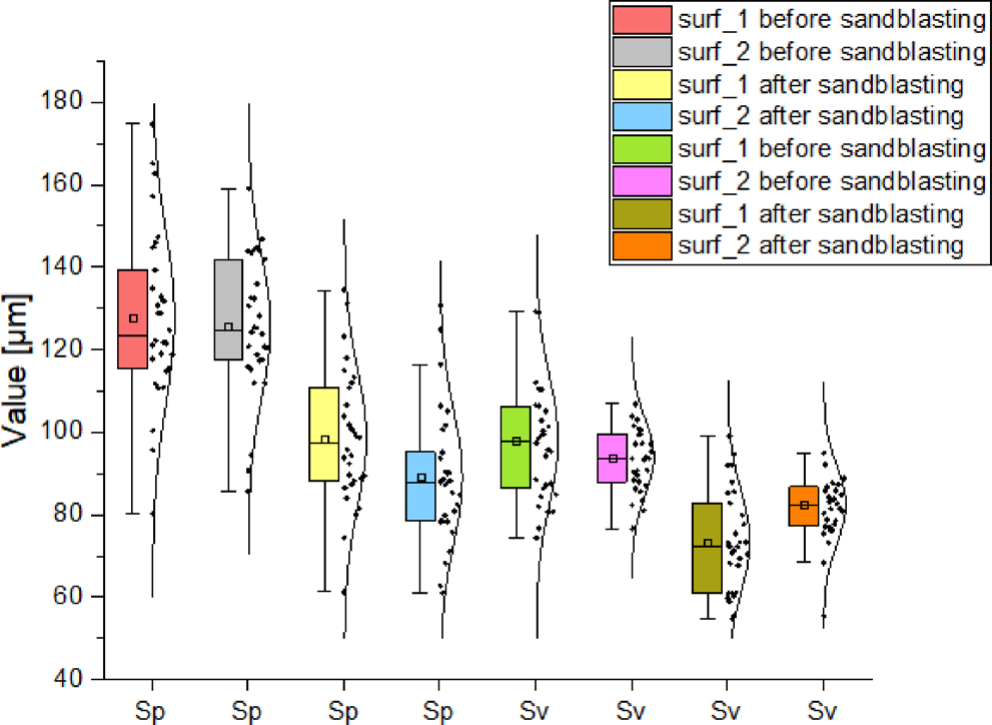
Distributions of the values of the parameters *Sp* and *Sv*: including the distribution of quartiles.

Figure 23 presents the distribution of the *Sp* and *Sv* parameter values for surfaces 1 and 2 for each sample. The whiskers indicate the standard deviation (*Sd*), while the box range represents the mean values. Additionally, the distribution of individual measurement values is shown, with indications of where each measurement occurred. The Sd values for all cases are at a similar level, indicating low measurement dispersion. This also confirms the normal distribution of the presented curves. A high level of the *Sp* parameter (representing prominent peaks) was observed before sandblasting for both surface 1 and surface 2 (*Sp* at approximately 130 μm). After post-processing, this value was reduced to around 90 μm. The drop in *Sp* values indicates a smoothing of the surface prominent protrusions were flattened. A similarly high value was observed for the *Sv* parameter (deep valleys), reaching around 100 μm before sandblasting. After treatment, this value decreased to approximately 80 μm. The change in *Sv* indicates an overall leveling of the surface, including both valleys and peaks. Although the abrasive reached recessed areas, the relative depth of valleys decreased due to the smoothing effect and the lowering of the mean surface plane.This demonstrates that the grit size was appropriately selected it allowed the treatment of the entire surface area. The *Sv* reduction indicates shallower valleys and an overall smoother surface. Importantly, the *Sp* value after sandblasting fell below the *Sv* value prior to sandblasting. This means that abrasive treatment reduced the tall peaks to a level even lower than the previous valleys. Furthermore, the difference between *Sp* and *Sv* after sandblasting is significantly smaller compared to before treatment. This suggests that the surface became more uniform, less rough, and better smoothed. This is further confirmed in Fig. 23, where the mean and median values for each pass of each sample were reduced. A point distribution of parameter values for individual samples, along with their density, is presented. The normal appearance of the distributions, inclusion of results within the appropriate percentile intervals, and clustering of values all support the validity of the data. From the graphs, it can be observed that *Sp* and *Sv* values for both surfaces before and after sandblasting are at comparable levels. Surface 1 exhibits slightly higher values (similar to the *Sa* and *Sq* parameters), yet the proportional relationship remained unchanged after sandblasting. After sandblasting, both *Sp* and *Sv* values were significantly reduced, with *Sp* values dropping below the pre-treatment *Sv* levels. In addition, the mean and median values of the *Sz* parameter representing the total vertical range of surface irregularities and closely related to *Sp* and *Sv* were significantly reduced by approximately 50 μm. The lower *Sz* values confirm earlier conclusions that both prominent peaks (*Sp*) and deep valleys (*Sv*) were leveled out.

**Fig. 24:**
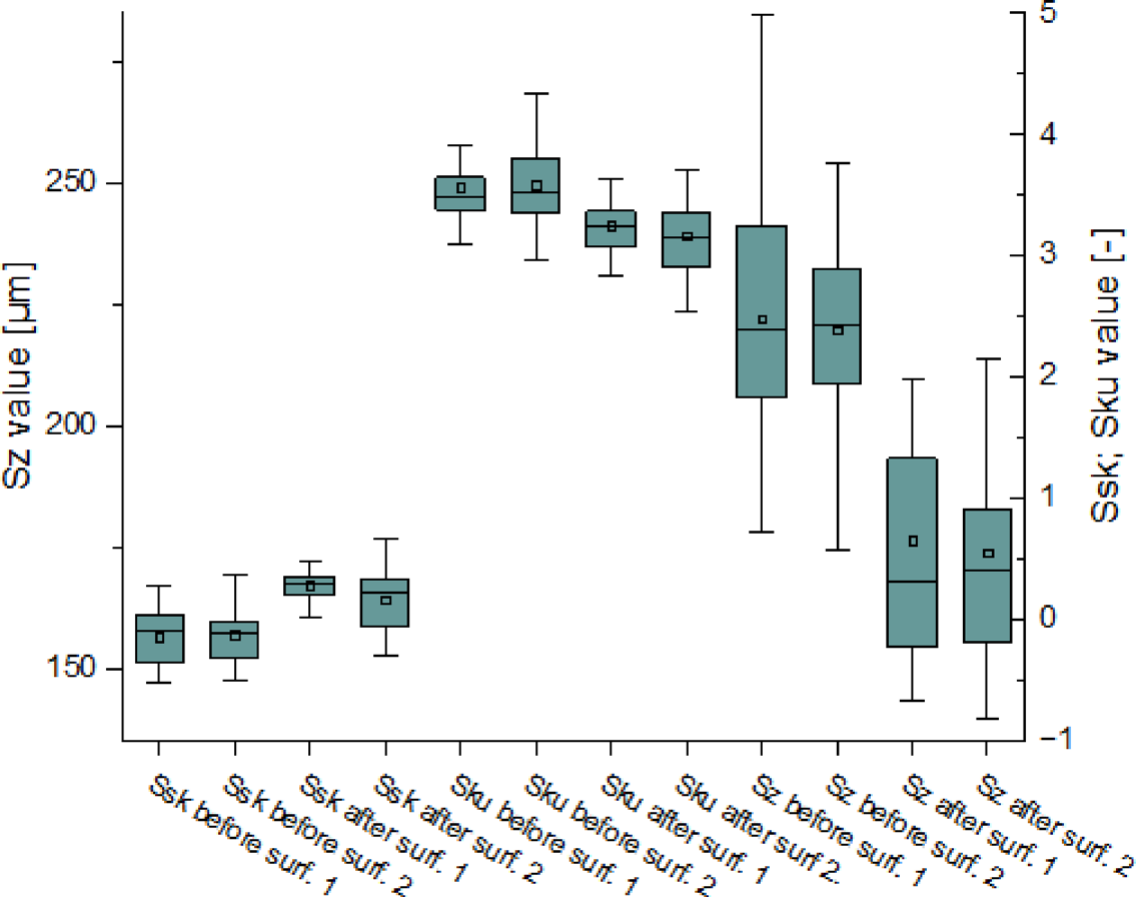
Distributions of *Ssk*, *Sku* and *Sz* parameter values ​​along with the trend and dependence of mutual changes.

Figure 24 presents the skewness (*Ssk*) – the asymmetry of the value distribution, kurtosis (*Sku*) – the peakedness of the distribution, and the total height of the surface profile (*Sz*). The *Sku* values before post-processing exceeded 3, indicating a leptokurtic distribution with many sharp peaks. These extreme values suggest the presence of sharp elevations and deep depressions on the surface. After sandblasting, the *Sku* values decreased and stabilized around 3, which suggests a shift towards a normal distribution. The reduction in extreme values indicates that the surface became more averaged and smoother, reflecting an overall homogenization of the surface texture. In turn, analysis of the changes in *Ssk* values shows a change in the sign of this parameter. Initially, *Ssk* values were below 0 (negative skewness), indicating a predominance of valleys over peaks. After sandblasting, this trend reversed: *Ssk* values became positive, suggesting that surface peaks became more prevalent than valleys. This trend, beyond being visible in Fig. 24, is also observable in the topographic views presented in Fig. 20. The reduction in the absolute values of these parameters also suggests a more uniform distribution of surface irregularities. In Fig. 24, one can observe the convergence of the *Ssk* and *Sku* parameter values, which indicates a significant change in the surface characteristics following post-processing, leading to a more uniform texture. Additionally, the figure highlights mutual relationships between *Sz*, *Sku*, and *Ssk*. It should be noted that *Sz*, *Sku*, and *Ssk* are amplitude parameters that describe roughness, meaning short-wavelength changes in the microstructure. However, based on the joint interpretation of *Sz*, *Sku*, and *Ssk*, it is also possible to assess the waviness i.e., the larger-scale, long-wavelength characteristics of the surface. If *Sz* has decreased, *Sku* has stabilized around 3, and *Ssk* has changed from negative to positive, this suggests that fine-scale roughness has been significantly reduced, but macroscopic elevations have become more pronounced. The distribution is now dominated by flatter, lower peaks rather than sharp, deep valleys. Following post-processing, the surface became less rough (as indicated by lower *Sz* and normal *Sku*), but its macro-waviness became more evident longer, smoother elevations are now more visible. Previously, these features were hidden beneath sharp micro-roughness. Fine roughness was diminished, and the longer, smoother waviness became more distinguishable. The reduction of *Sz*, *Sku* oscillating around 3, and positive *Ssk* all suggest that the surface waviness became more readable smoother, yet more pronounced waves emerged.

**Fig. 25:**
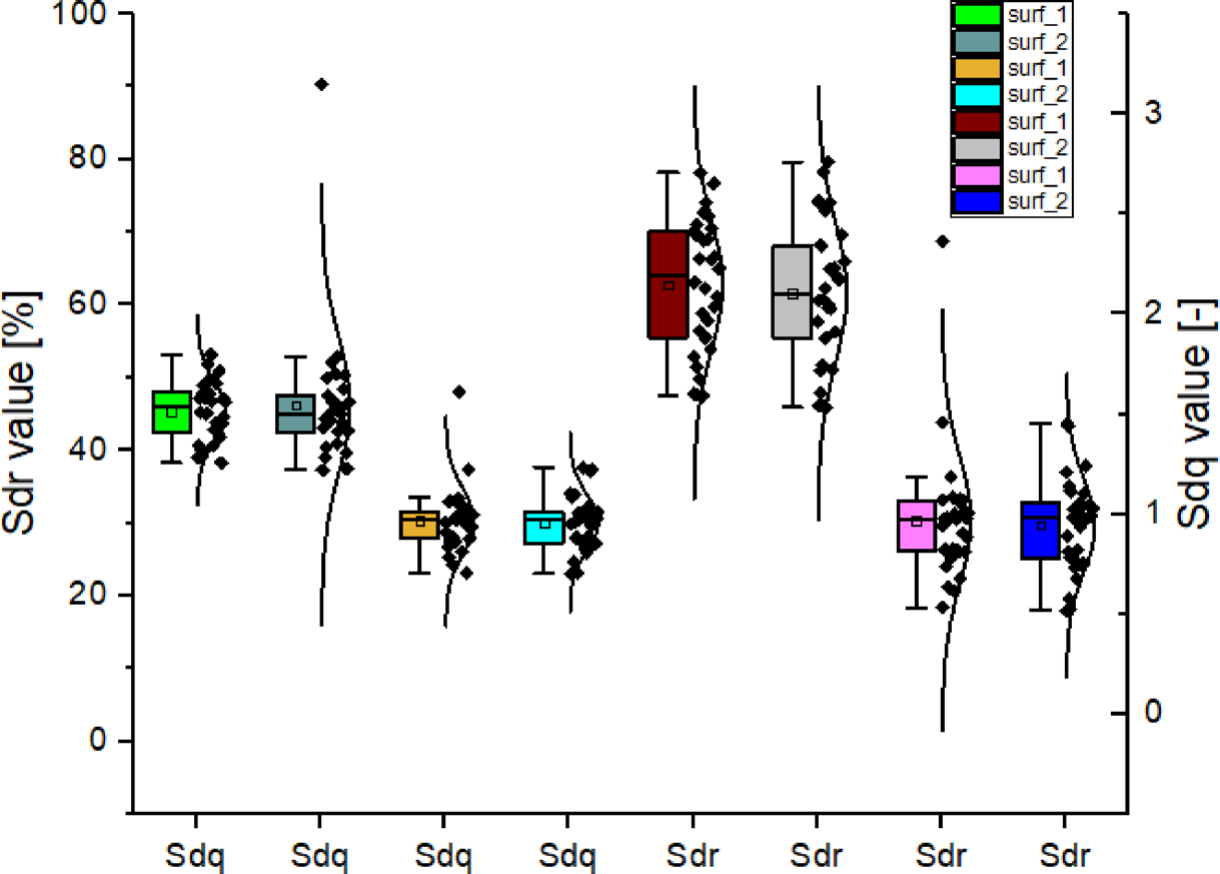
Distributions of the *Sdq* and *Sdr* parameter values.

Figure 25 presents the relationships involving the Developed Interfacial Area Ratio (*Sdr*) – which defines how much larger the actual surface area is compared to the projected (flat) area – and the Root Mean Square Slope (*Sdq*), which quantifies the average steepness of surface irregularities. The distributions indicate that *Sdr* consistently remains at a significantly higher level than *Sdq*, suggesting that the surfaces are rich in micro-irregularities but with low inclination angles and a low degree of waviness. After post-processing, both parameters show a notable decrease. The approximately twofold reduction in the values of *Sdq* and *Sdr* indicates that the surfaces became smoother and less complex. This signifies a substantial change in the geometric nature of the surface, which must be taken into account during the design and manufacturing processes. A general decline in both *Sdq* and *Sdr* values was observed after sandblasting. In the vast majority of samples, a reduction in *Sdq* was recorded, suggesting a smoothing of the surface’s micro-slopes. A decrease in *Sdr* was also observed, indicating flattening and simplification of the surface topography. In some isolated cases, *Sdr* values did not significantly change after sandblasting, which may point to local variability in the sandblasting effect due to the highly irregular and non-repetitive nature of the initial microstructure. Nevertheless, both *Sdq* and *Sdr* clearly decreased after post-processing, suggesting a reduction in the steepness of local irregularities and a decline in the developed surface structure. Additionally, the significant decrease in these parameters confirms that post-processing effectively smoothed out steep inclines and reduced the overall developed roughness of the surfaces.

**Fig. 26:**
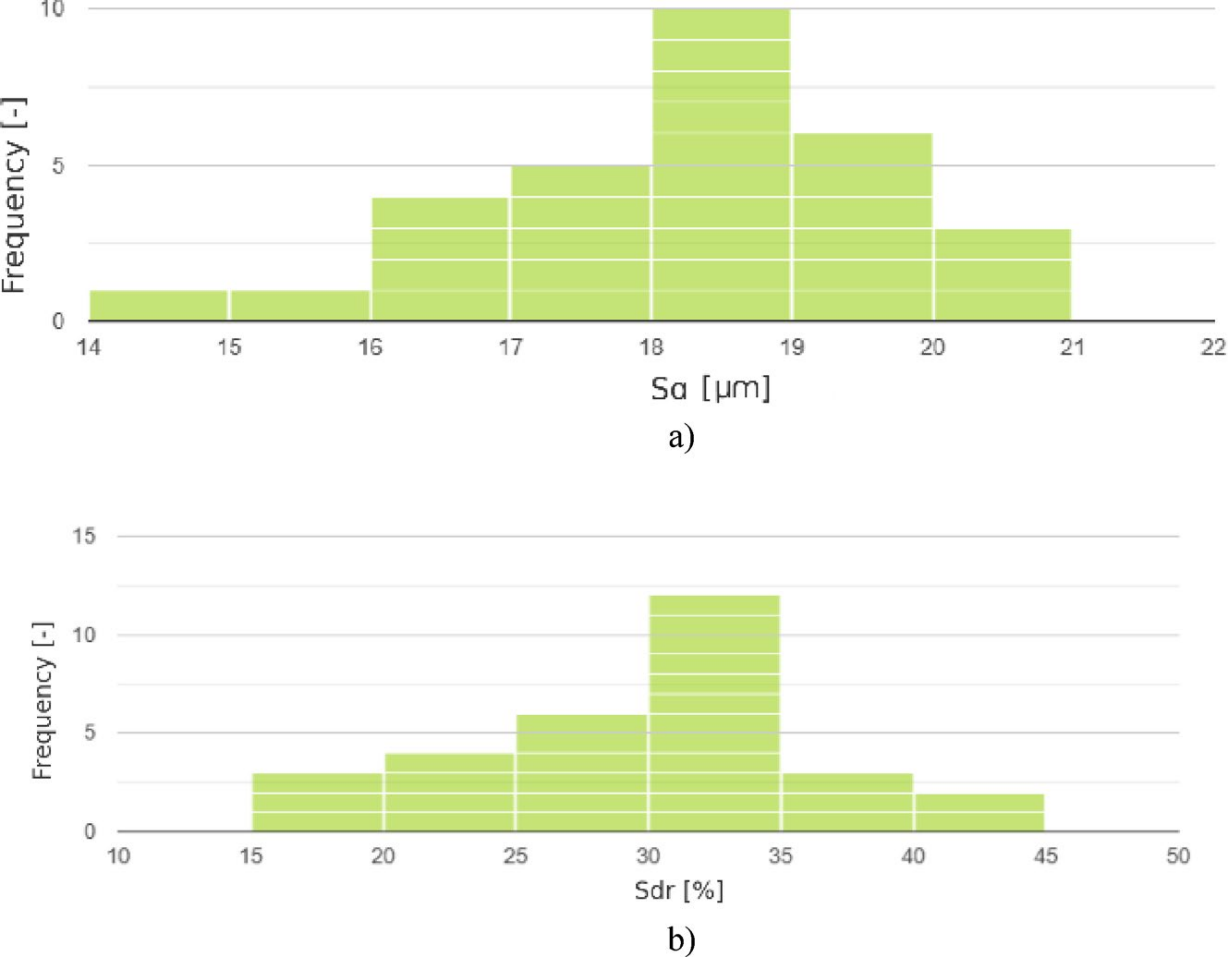
Example histograms of the distribution of values: (**a**) of the *Sa* parameter for surface 2 before sandblasting; (**b**) of the *Sdr* parameter for surface 2 after sandblasting.

Additionally, a statistical analysis was carried out for the obtained surface roughness parameters in accordance with ISO 25,178. For each measurement group and for each parameter, both before and after sandblasting, the Shapiro–Wilk test was performed. This allowed for verification of whether the obtained results followed a normal distribution. In total, 36 tests were conducted, resulting from the analysis of 9 parameters across two surface types from each sample, both before and after post-processing. The analysis showed that for each parameter, the p-value obtained was greater than 0.05. This means that the deviations from normality are not statistically significant, and the null hypothesis that the data are normally distributed is accepted. Sample histograms of parameter distributions are presented in Fig. 26.

After confirming the normality of the distributions for individual parameters, it was possible to perform the Student’s t-test. Since the same surfaces were measured both before and after post-processing, the test was used to determine whether the difference between the two measurements (pre- and post-sandblasting) was statistically significant. All previously measured surfaces and the corresponding surface roughness parameters were re-evaluated for this purpose. If the result of the t-test is statistically significant (*p* < 0.05), it indicates that there is a probable difference between the compared groups and that the observed change is not due to random variation. In the conducted analysis, for each group of parameters and surfaces, the obtained p-values were below 0.05. Therefore, it can be conclusively stated that the results obtained after sandblasting of MJF-manufactured surfaces significantly affect the surface characteristics and are statistically significant. What remained was to assess the magnitude of these effects. Ultimately, understanding the effect size after performing the t-test is crucial. To this end, the statistical effect size was calculated using Cohen’s D test, which estimates the strength of the difference between the two studied groups i.e., the surfaces before and after sandblasting. According to the interpretation of Cohen’s D, a value close to 0.2 indicates a small effect, around 0.5 a medium effect, and 0.8 or greater a large effect. The obtained results are presented in Table 8. For both Surface 1 and Surface 2, all Cohen’s D values exceed 0.8, indicating a large or very large effect of sandblasting on surface topography. The most pronounced changes were observed in parameters describing surface microstructure complexity and slope (*Sdq*, *Sdr*), clearly indicating effective surface smoothing. The smallest though still large effects were noted for the *Sku* and *Ssk* parameters, which may suggest that while sandblasting reduces extreme values, it does not completely eliminate them.

In addition, the Shapiro-Wilk test did not reveal any significant deviations from the normal distribution for any of the analyzed roughness parameters – in all cases, p-values exceeding the accepted significance level of 0.05 were obtained. This means that the distributions of measurements can be considered normal and meet the assumptions necessary for the use of parametric tests. These conclusions are confirmed by the analysis of the *Sku* kurtosis coefficient values presented in Fig. 24. This parameter, which describes the shape of the surface height distribution, takes a value close to 3 for a normal distribution. In the tested samples, the *Sku* values oscillate around this limit both before and after sandblasting, which indicates that the distribution is neither clearly peaked nor flattened. The convergence of the results of both assessment methods – the Shapiro-Wilk statistical test and the analysis of the *Sku* value – provides consistent and unambiguous confirmation that the obtained measurement data can be considered to be in accordance with the normal distribution, and any deviations are only random and statistically insignificant.

**Table 8:** Cohen’s D test results for selected roughness parameters in accordance with ISO 25,178.

Surface	Parameter								
	*Sq*	*Sa*	*Sp*	*Sv*	*Ssk*	*Sku*	*Sz*	*Sdq*	*Sdr*
1.	1.8204	1.7359	1.5842	1.8076	2.3566	1.1025	1.8077	3.6219	3.6631
2.	2.4365	1.7351	2.2313	1.2960	1.2356	1.3685	2.0897	2.3140	3.9791

### Chemical measurement

**Fig. 27:**
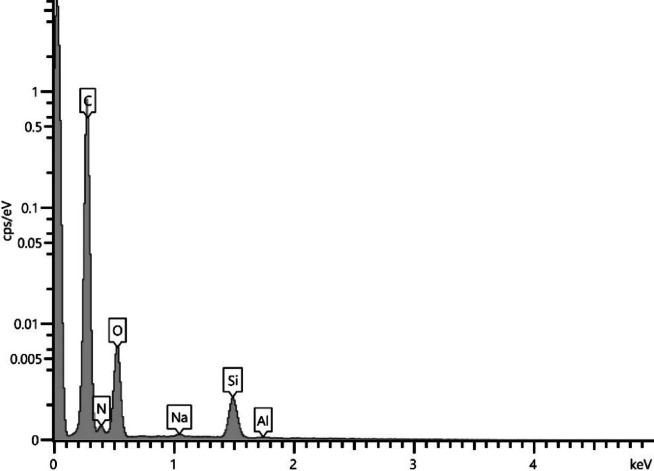
Elemental composition graph of keV vs. cps per keV through EDS analysis for sample 26.

Upon analyzing the keV elemental composition graph in terms of cps per keV using EDS analysis, we observe the presence of three main elements comprising the composition of the abrasive utilized during post-processing (Fig. 27). Fifteen independent measurements were taken at different surface locations for different samples. A rectangular area measuring 200 μm × 200 μm was scanned. The results for each sample were very similar – almost identical. A small proportion of sodium and silicon content has been observed, along with residual aluminum. No other elements present in the abrasive, but absent in the chemical composition of nylon, were detected. Furthermore, the cps/eV value for all the aforementioned elements, falling below 0.004, indicates a residual and negligible contribution of these elements to the chemical composition analysis. This is particularly notable for silicon, which constitutes 75% of the abrasive composition. Based on the findings, it can be deduced that residual abrasive remnants left after the post-processing operation were not considered in the surface analysis. The chemical composition of the samples does not suggest otherwise. In simpler terms, only the actual surface resulting from MJF printing and sandblasting was measured, excluding any possible inclusions resulting from beads on the sample surface.

### Discussion

The results of this study provide a comprehensive perspective on the influence of sandblasting on MJF-manufactured surfaces, particularly when analyzed through µCT imaging and multiscale surface characterization. The observed transformation of surface geometry—including a notable reduction in both roughness and the prevalence of re-entrant features – corresponds well with previously reported findings in additive manufacturing (AM), such as those by Singh et al.^57^ and Bartkowiak et al.^22^, who also reported surface smoothing after bead blasting or mechanical finishing. However, the magnitude of reduction observed here (Sa from 22.3 μm to 14.6 μm) exceeds values typically reported for laser-sintered polymers, suggesting a stronger smoothing effect linked to the lower hardness and ductility of PA12 compared to metal alloys used in other PBF processes.

The quantification of re-entrant features using newly defined metrics (*r₁*,* r₂*) represents a novel analytical contribution, expanding beyond traditional roughness analysis. These geometrical features – often invisible in 2D profilometry – were reduced by almost 80% after sandblasting, confirming the efficiency of the process. Similar reductions were noted qualitatively by Houngkamhang et al.^54^, yet without numerical characterization. This study therefore bridges that gap by providing measurable, quantitative evidence supported by µCT-based 3D reconstructions.

From a physical standpoint, the observed smoothing can be attributed to localized microplastic deformation and fracture of surface asperities under the impact of glass beads. The kinetic energy of abrasive particles leads to rounding of peaks and partial closure of fine pores. At the same time, due to the comparable size of the abrasive and polymer powder grains (~ 60 μm), the removal process remains controlled, avoiding deep erosive wear or subsurface damage. These mechanisms align with surface engineering models of solid-particle erosion described by Hutchings^58^ and subsequent AM-specific studies^24,44^.

The simplification of surface geometry is not purely morphological—it directly influences surface functionality. Previous works by Yamaguchi^51^ and Houngkamhang et al.^54^ emphasized that re-entrant microstructures affect adhesion, wettability, and tribological behavior. In this context, the present study identifies a potential functional trade-off: while sandblasting improves smoothness and dimensional precision, it can also suppress microscale features that enhance hydrophobicity or biocompatibility. Thus, the balance between functional roughness and form uniformity must be carefully tuned depending on the target application. For biomedical parts, smoother surfaces may minimize tissue irritation and bacterial adhesion, whereas in self-cleaning or friction-modulated components, preserving controlled micro-roughness could be beneficial. It should also be noted that recent studies^59^ have shown that doubly re-entrant geometries can exhibit superhydrophobicity, which not only promotes self-cleaning but also reduces bacterial adhesion through an antibiofouling mechanism. Although the re-entrant features generated by MJF and subsequently modified by sandblasting are random and lack the well-defined hierarchical geometry of engineered superhydrophobic surfaces, they may still contribute to partial antibiofouling effects depending on local geometry and surface chemistry. Future studies should therefore investigate how the stochastic nature of these additively manufactured re-entrant microstructures influences their biological performance, particularly in biomedical and hygienic applications.

The geometric characteristics of a surface directly affect the precision of part fitting, interaction at contact points, stress distribution, and frictional properties. In precision assemblies, bearing systems, or structural joints, deviations in surface geometry can cause clearance, poor load transfer, or premature wear. Hence, the reduced variability and enhanced uniformity observed post-sandblasting have direct implications for dimensional control and fatigue performance in AM components.

The µCT-based approach presented here extends the analytical scope beyond conventional profilometry by enabling volumetric quantification of sub-surface and re-entrant features. While earlier studies by Bartkowiak et al.^22^ used µCT primarily for defect detection and Brown et al. for multiscale analysis^44^, the present work demonstrates its metrological applicability. The integration of multiscale metrics (*Asfc*, *Srel*) further refines this understanding, aligning with Mandelbrot’s fractal surface theory^45^ and showing how complexity decreases across scales below ~ 10,000 μm² after sandblasting.

The use of sodium-silica glass beads with slightly larger particle size than PA12 powder ensured efficient powder removal without embedding or contamination, as confirmed by SEM-EDS and µCT analysis. These results agree with previous reports^10,29^, but the combination of chemical and tomographic verification applied here strengthens confidence in the post-processing integrity.

From a manufacturing perspective, these findings provide guidance for optimizing MJF workflows. Surface texture is known to affect fatigue life, stress initiation, and interfacial bonding^23^. The current results demonstrate that the final part quality depends on the combined effects of printing and finishing. Therefore, post-processing must be integrated into predictive models and tolerance definitions for functional components in biomedical, aerospace, and electronic systems.

Statistical analysis supported these conclusions: the Shapiro–Wilk test confirmed data normality, while the Student’s t-test and Cohen’s d indicated very large effect sizes (d > 1.0), particularly for *Sdq* and *Sdr*, reflecting pronounced microstructural simplification. The consistent trends across both analyzed surfaces demonstrate the uniformity and repeatability of sandblasting.

Although effective, the process is sensitive to local jet pressure and exposure time, which can introduce minor variations. Future studies should aim to model these effects and optimize sandblasting parameters through data-driven or machine-learning methods using µCT feedback. Such predictive approaches could establish direct correlations between build orientation, local geometry, and post-processing response, enabling improved process control in industrial AM.

## Conclusion

This study provides a detailed analysis of surfaces produced using the Multi Jet Fusion (MJF) 3D printing method, before and after sandblasting. The use of micro-computed tomography (µCT), ISO roughness parameters, and multiscale geometric analysis enabled comprehensive evaluation of surface morphology, including re-entrant features. µCT proved to be the only technique capable of full 3D reconstruction and quantitative assessment of these geometries, impossible with conventional profilometric methods.

Sandblasting with sodium–silicate glass beads (~ 60 μm) effectively removed unsintered powder without contaminating or damaging the PA12 surface, which was confirmed by chemical and tomographic analyses. Post-processing significantly reduced re-entrant features – the *r₂* parameter (surface area fraction) decreased from 60% to 13%, while *r₁* (relative re-entrant surface area) dropped from 260% to 135%, indicating effective elimination of closed cavities.

Surface roughness parameters *Sa*, *Sq*, *Sp*, and *Sv* were considerably reduced, confirming homogenization and smoothing of the microgeometry. The mean Sa decreased to ~ 15 μm, and *Sq* showed a similar trend. *Sp* fell from ~ 130 μm to 90 μm and *Sv* from ~ 100 μm to 80 μm, demonstrating the removal of peaks and partial leveling of depressions. The *Sz* parameter was also reduced, while *Ssk* and *Sku* approached values typical of a normal distribution (*Ssk* ≈ 0, *Sku* ≈ 3). Lower standard deviations of all parameters indicated greater surface uniformity and repeatability. Statistical tests (Shapiro–Wilk, Student’s t-test, Cohen’s d) confirmed the significance of these changes.

Multiscale geometric analysis further showed that the most pronounced differences between pre- and post-sandblasted surfaces occur below 10,000 μm². The relative surface area (*Srel*) and complexity (*Asfc*) were clearly reduced, confirming smoothing at fine scales. Surfaces after post-processing exhibited more regular and repeatable textures, with decreased coefficient of variation (CV) and statistically significant differences (MSR > 6.85, *p* < 0.001).

Overall, sandblasting substantially improved surface quality by reducing re-entrant structures, irregularities, and roughness. These findings highlight the importance of controlling post-processing parameters in MJF manufacturing, particularly for precision, mechanical, and biomedical applications where surface integrity is critical.

## Data Availability

The acquired data have been deposited in the Zenodo repository: 10.5281/zenodo.16902088.

## References

[1] Perez-Barcenilla, S. et al. Applicability of a material constitutive model based on a transversely isotropic behaviour for the prediction of the mechanical performance of multi jet fusion printed polyamide 12 parts. *Polymers* **16**, (2024). 10.3390/polym16010056 PMC10780423 38201721

[2] Liu, Z., Zhang, P., Yan, M., Xie, Y. & Huang, G. Additive manufacturing of specific ankle-foot orthoses for persons after stroke: A preliminary study based on gait analysis data. *Math. Biosci. Eng.* **16**, 8134–8143 (2019). 31698659 10.3934/mbe.2019410

[3] Binedell, T., Meng, E. & Subburaj, K. Design and development of a novel 3D-printed non-metallic self-locking prosthetic arm for a forequarter amputation. *Prosthet. Orthot. Int.* **45**, 94 (2021). 33834751 10.1177/0309364620948290

[4] Oh, S. A. et al. Fabrication of a patient-customized helmet with a three-dimensional printer for radiation therapy of scalp. *Prog Med. Phys.* **28**, 100 (2017).

[5] Wittkopf, J. A. et al. 3D printed electronics with multi jet fusion. **2019-September** 29–33 (2019).

[6] Wittkopf, J. A. et al. 3Dprinted electronics with multi jet fusion for flexible hybrid electronics. In *2023 IEEE 73rd Electronic Components and Technology Conference (ECTC)* 1463–1470 10.1109/ECTC51909.2023.00249 (2023).

[7] Li, M. et al. Enhanced heat transfer in 3D printed ball-end grinding tool with blade-shaped structure. *Appl. Therm. Eng.* **244**, (2024).

[8] Kajzer, W. et al. Tests of threaded connections made by additive manufacturing technologies. In *Innovations in Biomedical Engineering* (eds Tkacz, E. et al.) 329–337 10.1007/978-3-030-15472-1_35. (Springer International Publishing, 2019).

[9] Haq, M. R., ul, Nazir, A., Lin, S. C. & Jeng, J. Y. Investigating the effect of design parameters on the mechanical performance of contact wave springs designed for additive manufacturing. *3D Print. Additive Manuf.* **11**, 94–114 (2024). 10.1089/3dp.2021.0313 PMC10880676 38389690

[10] Shah, G. J., Haq, M. R., ul, Lin, S. C. & Jeng, J. Y. Analysis of variable frictional contacts wave springs fabricated using multijet fusion additive manufacturing. *Int. J. Adv. Manuf. Technol.* **126**, 87–101 (2023).

[11] Bhat, C., Kumar, A., Lin, S. C. & Jeng, J. Y. Adaptive mechanical properties and stretchability of novel chainmail fabrics based on overlapping tessellation strategies. *Additive Manuf.* **76**, 103777 (2023).

[12] Dizon, J. R. C., Gache, C. C. L., Cascolan, H. M. S., Cancino, L. T. & Advincula, R. C. Post-processing of 3D-printed polymers. *Technologies* **9**, 61 (2021).

[13] Hardiman, K. Post-processing considerations for biomedical 3D printing of polymers. In *Polymer-Based Additive Manufacturing: Biomedical Applications* (ed Devine, D. M.) 219–241 10.1007/978-3-030-24532-0_10. (Springer International Publishing, 2019).

[14] Peng, X., Kong, L., Fuh, J. Y. H. & Wang, H. A review of post-processing technologies in additive manufacturing. *J. Manuf. Mater. Process.* **5**, 38 (2021).

[15] Tamburrino, F., Barone, S., Paoli, A. & Razionale, A. V. Post-processing treatments to enhance additively manufactured polymeric parts: a review. *Virtual Phys. Prototyp.* **16**, 221–254 (2021).

[16] Hays, D. F. Squeeze films for rectangular plates. *J. Basic Eng.* **85**, 243–246 (1963).

[17] Vulliez, M. et al. Multi-scale curvature analysis and correlations with the fatigue limit on steel surfaces after milling. *Procedia CIRP*. **13**, 308–313 (2014).

[18] Bartkowiak, T., Berglund, J. & Brown, C. A. Establishing functional correlations between multiscale areal curvatures and coefficients of friction for machined surfaces. *Surf. Topogr : Metrol. Prop.* **6**, 034002 (2018).

[19] Li, Y., Alame, K. & Mahesh, K. Feature-resolved computational and analytical study of laminar drag reduction by superhydrophobic surfaces. *Phys. Rev. Fluids*. **2**, 054002 (2017).

[20] Khorasani, M. et al. Multi jet fusion (MJF) of polymeric components: A review of process, properties and opportunities. *Additive Manuf.* **91**, 104331 (2024).

[21] Ju, A. et al. Improving aesthetics through post-processing for 3D printed parts. *Electron. Imaging*. **31**, 1–5 (2019).

[22] Bartkowiak, T., Gapiński, B., Wieczorowski, M., Mietliński, P. & Brown, C. A. Capturing and characterizing geometric complexities of metal additively manufactured parts using x-ray micro-computed tomography and multiscale curvature analyses. *Surf. Topogr : Metrol. Prop.* **11**, 014002 (2023).

[23] Thompson, A., Senin, N., Giusca, C. & Leach, R. Topography of selectively laser melted surfaces: A comparison of different measurement methods. *CIRP Ann.* **66**, 543–546 (2017).

[24] Key, A. et al. Quantifying edge sharpness on stone flakes: comparing mechanical and micro-geometric definitions across multiple raw materials from Olduvai gorge (Tanzania). *J. Archaeol. Method Theory*. **31**, 51–74 (2024).

[25] Bartkowiak, T. et al. Wetting properties of polymer additively manufactured surfaces – multiscale and multi-technique study into the surface-measurement-function interactions. *Tribol. Int.* **202**, 110394 (2025).

[26] Whitehouse, D. J. *Handbook of Surface Metrology*10.1201/9780203752609 (Routledge, 2023).

[27] Leach, R. *Optical Measurement of Surface Topography*. *Optical Measurement of Surface Topography by Richard Leach. Berlin: Springer*, ISBN: 978-3-642-12011-4 10.1007/978-3-642-12012-1 (2011).

[28] Guo, B., Xu, Z., Luo, X. & Bai, J. A detailed evaluation of surface, thermal, and flammable properties of polyamide 12/glass beads composites fabricated by multi jet fusion. *Virtual Phys. Prototyping* **16**, S39–S52 (2021).

[29] Tey, W., Cai, C. & Zhou, K. A comprehensive investigation on 3D printing of polyamide 11 and thermoplastic polyurethane via multi jet fusion. *Polymers* **13**, 2139 (2021). 34209747 10.3390/polym13132139 PMC8271651

[30] Chand, R., Sharma, V. S. & Trehan, R. Investigating mechanical properties of 3D printed parts manufactured in different orientations on multijet printer. *Int. J. Mechatronics Manuf. Syst.* **14**, 164–179 (2021).

[31] Sagbas, B., Gümüş, B. E., Kahraman, Y. & Dowling, D. P. Impact of print bed build location on the dimensional accuracy and surface quality of parts printed by multi jet fusion. *J. Manuf. Process.* **70**, 290–299 (2021).

[32] Tasch, D., Schagerl, M., Wazel, B. & Wallner, G. Impact behavior and fractography of additively manufactured polymers: laser sintering, multijet fusion, and hot lithography. *Additive Manuf.* **29**, 100816 (2019).

[33] Chen, J., Zhao, L. & Zhou, K. Improvement in mechanical performance of the multi jet fusion–printed aramid fiber/polyamide 12 composites by fiber surface modification. *Additive Manuf.* **51**, 102576 (2021).

[34] Alomarah, A., Abbas, A., Faisal, B., Peng, Z. & Ruan, D. The effects of manufacturing techniques on the mechanical performance of an auxetic structure manufactured by fused filament fabrication and multijet fusion processes. *Adv. Eng. Mater.* **26**, (2024).

[35] Townsend, A., Senin, N., Blunt, L., Leach, R. K. & Taylor, J. S. Surface texture metrology for metal additive manufacturing: a review. *Precis. Eng.* **46**, 34–47 (2016).

[36] Mirabal, A. et al. Roughness measurements across topographically varied additively manufactured metal surfaces. *Additive Manuf.* **69**, 103540 (2023).

[37] Mason, B., Kundu, A., Ryan, M., Setchi, R. & Bhaduri, D. A robust methodology for surface roughness characterisation of additive manufactured parts. *Proc. Institution Mech. Eng. Part. L: J. Materials: Des. Appl.*10.1177/14644207251353815 (2025).

[38] ISO 25178-2. 2012(en), Geometrical product specifications (GPS) — Surface texture: Areal — Part 2: Terms, definitions and surface texture parameters. https://www.iso.org/obp/ui/#iso:std:iso:25178:-2:ed-1:v1:en.

[39] Sillani, F., Kleijnen, R. G., Vetterli, M., Schmid, M. & Wegener, K. Selective laser sintering and multi jet fusion: Process-induced modification of the raw materials and analyses of parts performance. *Additive Manuf.* **27**, 32–41 (2019).

[40] Development Company, H. P. L. P. HP 3D High Reusability PA 12 Glass Beads. (2019).

[41] B46.1 Surface Texture Quality - ASME. https://www.asme.org/codes-standards/find-codes-standards/b46-1-surface-texture.

[42] Królczyk, J. B. et al. Review of surface metrology artifacts for additive manufacturing. *Bull. Pol. Acad. Sci. Tech. Sci.* **72**(6), e151380 (2024).

[43] Brown, C. A. Surface metrology principles for snow and ice friction studies. *Front. Mech. Eng* **7**, (2021).

[44] Brown, C. A. et al. Multiscale analyses and characterizations of surface topographies. *CIRP Ann.* **67**, 839–862 (2018).

[45] Ferson, S. The fractal geometry of Nature.Benoit B. Mandelbrot. *Q. Rev. Biol.* https://www.academia.edu/119829493/The_Fractal_Geometry_of_Nature_Benoit_B_Mandelbrot (1983).

[46] Peta, K., Stemp, W. J., Chen, R., Love, G. & Brown, C. A. Multiscale characterizations of topographic measurements on lithic materials and microwear using a gelsight max: investigating potential archaeological applications. *J. Archaeol. Science: Rep.* **57**, 104637 (2024).

[47] Peta, K. Multiscale wettability of microtextured irregular surfaces. *Materials* **17**, 5716 (2024). 39685152 10.3390/ma17235716 PMC11642355

[48] Macdonald, D. A. et al. Revisiting lithic edge characterization with microct: multiscale study of edge curvature, re-entrant features, and profile geometry on Olduvai gorge quartzite flakes. *Archaeol. Anthropol. Sci.* **14**, 33 (2022).

[49] Jakubowicz, M. et al. Parametric evaluation samples made by SLM technology measured using Micro-Computed tomography. in Advances in Manufacturing IV (eds (eds Diering, M., Wieczorowski, M. & Harugade, M.) 83–95 (Springer Nature Switzerland, Cham, doi:10.1007/978-3-031-56467-3_7. (2024).

[50] Peta, K., Mendak, M. & Bartkowiak, T. Discharge energy as a key contributing factor determining microgeometry of aluminum samples created by electrical discharge machining. *Crystals* **11**, 1371 (2021).

[51] Yamaguchi, M. Microfabrication of re-entrant surface with hydrophobicity/oleophobicity for liquid foods. *Sci. Rep.* **10**, 2250 (2020). 32042014 10.1038/s41598-020-59149-2 PMC7010658

[52] Sow, P. K., Singhal, R., Sahoo, P. & Radhakanth, S. Fabricating low-cost, robust superhydrophobic coatings with re-entrant topology for self-cleaning, corrosion inhibition, and oil-water separation. *J. Colloid Interface Sci.* **600**, 358–372 (2021). 34023697 10.1016/j.jcis.2021.05.026

[53] Lee, D. K., Lee, E. H. & Cho, Y. H. A superoleophobic surface with anisotropic flow of hexadecane droplets. *Microsyst. Technol.* **23**, 421–427 (2017).

[54] Houngkamhang, N. et al. Enhancement of bacterial anti-adhesion properties on robust PDMS micro-structure using a simple flame treatment method. *Nanomaterials* **12**, 557 (2022). 35159902 10.3390/nano12030557 PMC8839957

[55] ARENA French manufacturer of sandblasting cabins. https://arenablast.com/

[56] Peta, K., Love, G. & Brown, C. A. Comparing repeatability and reproducibility of topographic measurement types directly using linear regression analyses of measured heights. *Precis. Eng.* **88**, 192–203 (2024).

[57] Singh, P. K., Kumar, S. & Jain, P. K. Additive manufacturing technologies and post-processing, design optimization, and material considerations for reliable printing. In *Nanotechnology-Based Additive Manufacturing* 27–52 10.1002/9783527835478.ch2 (Wiley , 2023).

[58] Hutchings, I. *Mechanisms of the Erosion of Metals by Solid Particles* (ASTM International, 1979).

[59] Lee, M. S. et al. Superrepellent doubly reentrant geometry promotes antibiofouling and prevention of coronavirus contamination. *Adv. Mater. Technol.*10.1002/admt.202200387 (2022). 36247709 10.1002/admt.202200387 PMC9538020

